# Chromosomal Organization and Segregation in *Pseudomonas aeruginosa*

**DOI:** 10.1371/journal.pgen.1003492

**Published:** 2013-05-02

**Authors:** Isabelle Vallet-Gely, Frédéric Boccard

**Affiliations:** 1 CNRS, Centre de Génétique Moléculaire, UPR3404, Gif-sur-Yvette, France; 2 Université Paris-Sud, Orsay, France; Institute of Molecular and Cell Biology (IMCB), A*STAR, Singapore

## Abstract

The study of chromosomal organization and segregation in a handful of bacteria has revealed surprising variety in the mechanisms mediating such fundamental processes. In this study, we further emphasized this diversity by revealing an original organization of the *Pseudomonas aeruginosa* chromosome. We analyzed the localization of 20 chromosomal markers and several components of the replication machinery in this important opportunistic γ-proteobacteria pathogen. This technique allowed us to show that the 6.3 Mb unique circular chromosome of *P. aeruginosa* is globally oriented from the old pole of the cell to the division plane/new pole along the *oriC*-*dif* axis. The replication machinery is positioned at mid-cell, and the chromosomal loci from *oriC* to *dif* are moved sequentially to mid-cell prior to replication. The two chromosomal copies are subsequently segregated at their final subcellular destination in the two halves of the cell. We identified two regions in which markers localize at similar positions, suggesting a bias in the distribution of chromosomal regions in the cell. The first region encompasses 1.4 Mb surrounding *oriC*, where loci are positioned around the 0.2/0.8 relative cell length upon segregation. The second region contains at least 800 kb surrounding *dif*, where loci show an extensive colocalization step following replication. We also showed that disrupting the ParABS system is very detrimental in *P. aeruginosa*. Possible mechanisms responsible for the coordinated chromosomal segregation process and for the presence of large distinctive regions are discussed.

## Author Summary

The processes of chromosomal disposition, replication, and segregation in bacteria have been characterized only in a handful of species, yet there is remarkable diversity in the ways such fundamental processes are managed. In this study, we analyzed the subcellular chromosomal organization of *Pseudomonas aeruginosa*, an important bacterial pathogen belonging to a large bacterial group involved in plant and human diseases. Most bacterial genomes are circular molecules, and DNA replication proceeds bidirectionally from a single origin to the opposite Ter region, where the replication forks meet. Analysis by fluorescence microscopy of 20 chromosomal markers and components of the replication machinery revealed that the 6.3 Mb chromosome is globally oriented from the old pole of the cell to the division plane/new pole along the *oriC*-Ter axis. The replication machinery is positioned at mid-cell, and chromosomal loci from *oriC* to Ter are moved sequentially to mid-cell prior to replication. The two sister chromosomes are subsequently segregated at their final subcellular destination in the two halves of the cell. This study also identified two large regions in which several chromosomal loci show a biased localization pattern, suggesting that processes responsible for long-range chromosomal organization might exist in *P. aeruginosa*.

## Introduction

Members of the genus *Pseudomonas* (γ-proteobacteria) show remarkable metabolic and physiological diversity and versatility, enabling colonization of diverse terrestrial and aquatic habitats. These species are of great interest because of their importance in plant and human diseases and their growing potential in biotechnological applications. One of these bacteria, *Pseudomonas aeruginosa*, is an aquatic and soil bacterium that can infect a range of organisms, including plants, invertebrates and various mammals [1]. In humans, *P. aeruginosa* is an opportunistic pathogen that causes serious infections in immunocompromised patients, and it is the leading cause of morbidity in cystic fibrosis patients [2]. These infections are particularly challenging because of *P. aeruginosa's* broad intrinsic antimicrobial resistance [3].

The PAO1 genome was the first *P. aeruginosa* genome to be sequenced [4]. It comprises 6.3 Mbp encoding 5,570 genes. Its size results from genetic complexity rather than gene duplications, which may suggest that it adapts to colonize a diverse range of ecological niches [1], [4]. A comparison of five *P. aeruginosa* genomes revealed a relatively large set of 5021 conserved genes, which constitute the core genome. Beside this set, insertions containing blocks of strain-variable genes are found in a limited number of chromosomal locations, termed Regions of Genomic Plasticity (RGPs). The *P. aeruginosa oriC* sequence has been characterized and is adjacent to *dnaA* and *dnaN* [5], [6]. The *dif* site, a sequence that is required for chromosome dimer resolution by the XerCD recombinases upon activation by the DNA translocase FtsK, was identified opposite of *oriC*, whereas no system dedicated to replication termination (such as replication fork trap) has yet been characterized in *P. aeruginosa*.

To fit inside bacterial cells, bacterial chromosomes need to be organized into a compact structure called the nucleoid. DNA compaction is thought to result from the interplay between macromolecular crowding, DNA supercoiling and the specific action of DNA binding proteins [7]. In all organisms in which this process has been examined, the nucleoid is oriented in a specific way inside the cell, which preserves the linear order of genes in the DNA [8]. This global orientation is longitudinal in the case of *Bacillus subtilis*, *Caulobacter crescentus* and *Vibrio cholerae* chromosomes and transversal in *E. coli* [9], [10]. In *E. coli*, large-scale organization in several domains is also present [11], [12].

The compaction of the chromosome must be compatible with various processes of DNA metabolism, such as gene expression and replication and the segregation of genetic information. Different hypotheses have been proposed to explain chromosomal segregation. For instance, studies in *B. subtilis* led to the development of the capture extrusion model, which suggests that the energy required for initial chromosomal segregation could come from the replication process that occurs at mid-cell in a static replication factory [13]. However, it was later shown that in *E. coli*, the two replication forks appear to follow chromosomal arms, rendering the capture extrusion model irrelevant for *E. coli* chromosomes [14], [15]. It was proposed instead that loss of cohesion between replicated origin regions could trigger global chromosomal movement and mediate chromosomal segregation [14]. It has also been proposed that entropic exclusion of replicated chromosomes might participate in the segregation process [16].

At the molecular level, a mitotic-like apparatus composed of a DNA binding protein (ParB) that binds to a specific sequence (*parS*) and a Walker-type ATPase (ParA) is present in a number of bacteria, including *P. aeruginosa*, but absent in *E. coli*. This apparatus is involved in chromosomal segregation in *V. cholera*, *C. crescentus* (where it is essential), *B. subtilis* and *Streptococcus pneumonia* [17]–[20]. It was also demonstrated in *B. subtilis* and *S. pneumonia* that the recruitment of bacterial condensins to the origin region by ParB-*parS* complex contributes to chromosomal segregation [20]–[22]. Two bacterial condensins have been identified in *P. aeruginosa*, and both the ParABS system and these two condensins appear to be important for chromosomal segregation, as the deletion of either of these factors leads to an increase in anucleated cells [23]–[25].

As a first step in the study of chromosomal organization and segregation in *P. aeruginosa*, we used a fluorescent microscopy approach to investigate chromosomal localization during growth. This approach allowed us to show that the global orientation of the *P. aeruginosa* chromosome is longitudinal and that the replication forks are mostly colocalized near mid-cell. Chromosomal loci appear to be relocated closer to mid-cell prior to replication, and the two replicated copies are then progressively segregated into opposite cell halves. This process is surprisingly different from what is observed in *E. coli*, another gamma-proteobacterium, but closer to what has been described for the gram-positive bacterium *B. subtilis*. Moreover, we could discern two distinctive regions in the *P. aeruginosa* chromosome, one surrounding *oriC* and the other surrounding the *dif* site. The origin region was defined by the fact that approximately 1.4 Mbp of DNA centered on *oriC* segregate to the same position around the 0.2/0.8 relative cell length. The Ter region encompasses at least 800 kbp that remain close to the new pole of the cell before being relocated to near mid-cell prior to replication. Loci in the Ter region show a high level of colocalization following replication. Chromosomal arms linking these two regions are longitudinally distributed. These features allow us to propose *P. aeruginosa* chromosomal organization as an original model, combining longitudinal organization, as observed in *C. crescentus*, with large distinctive regions that might be suggestive of long-range organization, as observed in *E. coli*. We also provide evidence that the ParABS system plays a major role in chromosomal organization and segregation in *P. aeruginosa*.

## Results

### Visualizing 20 chromosomal loci in live *P. aeruginosa* cells

**Figure 1:**
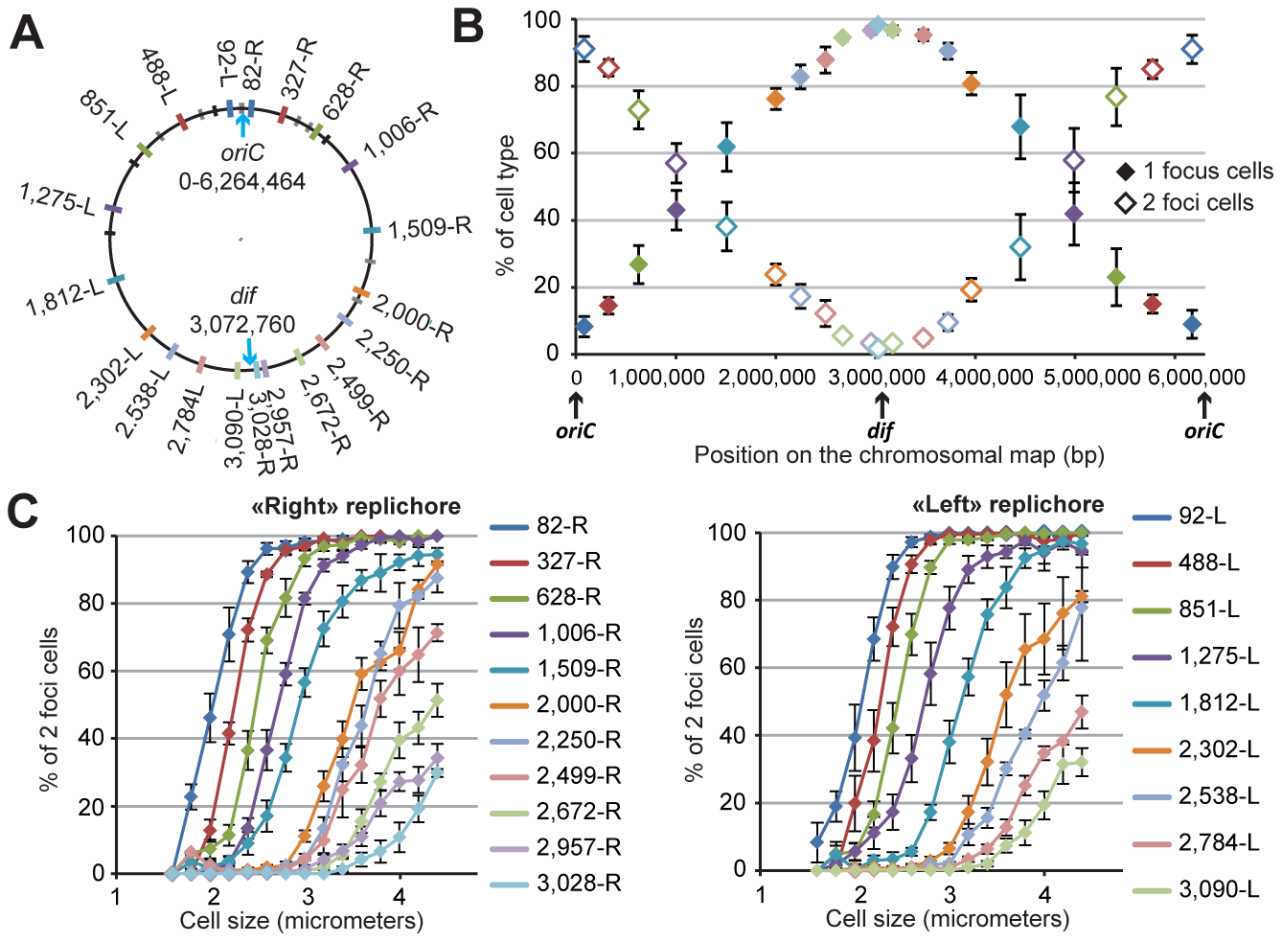
Progressive segregation of *P. aeruginosa* chromosomal loci. (A) Position of each chromosomal locus on the PAO1 chromosomal map. Each locus is represented by a colored tag on the PAO1 chromosome according to its position. The color code is used for all figures. Black tags indicate rRNA operons. Grey tags represent *parS* sequence positions. Four of these *parS* sites are clustered close to *oriC*, four more are dispersed between positions 851-L and 628-R, and two are localized in the “right” replichore. (B) Average percentage of one-focus cells (solid diamonds) and two-foci cells (open diamonds) in a bacterial population grown in minimal medium supplemented with citrate. Cells with no visible focus were removed from this analysis (they constituted between 5 to 10% of the cells). The proportion of cells exhibiting more than two foci was always smaller than 0.5 %. The *x*-axis represents the positions of the loci on the chromosomal map. Values from four to eleven experiments were averaged, and the error bars represent standard deviations. (C) The average percentage of two-foci cells in a bacterial population grown in minimal medium supplemented with citrate, according to cell size, for each chromosomal locus of the “right” replichore (left panel) or of the “left” replichore (right panel). Values from four to eleven experiments were averaged, and the error bars represent standard deviations.

To study chromosomal organization in *P. aeruginosa*, we used a fluorescent microscopy approach. The intracellular position of chromosomal loci was visualized using two different reporter systems based on the binding of a fluorescently labeled protein to a target sequence inserted at twenty specific locations in the *P. aeruginosa* chromosome (see Figure 1A and Text S1). We used both the TetR-CFP chimera, which recognizes *tetO* arrays, and the yGFP-ParB^pMT1^ chimera, which binds to the *parS^pMT1^* sequence [26], [27]. We created a plasmid allowing expression of both *yGFP-parB^pMT1^* and *tetR-CFP* from an IPTG inducible promoter. The visualization of two chromosomal loci by fluorescent microscopy was thus possible upon growth in IPTG-containing medium. The use of either the *parS^pMT1^* sequence or the *tetO* arrays to label a specific chromosomal locus gives similar results regarding foci number and positions inside the cell (data not shown).

### Chromosomal loci are sequentially segregated

We tested different growth conditions and chose to perform extensive studies in minimal medium supplemented with citrate at 30 degrees. Under these growth conditions, the *P. aeruginosa* doubling time is approximately 45 to 50 minutes, with cell sizes ranging from 2 to 5 µm (mean size of approximately 2.9 µm). We predicted, based on the number of foci corresponding to chromosomal loci or replisome proteins (see below), that most of the cells would contain a single replicating chromosome. Indeed, each chromosomal locus is visualized as one or two foci. Figure 1B shows the number of cells exhibiting one or two foci for each of the 20 positions. Loci close to *oriC* are mostly visualized as two foci (>90% of two-foci cells), indicating that they are replicated and segregated upon cell birth (focus duplication corresponds to the segregation of the two copies of a chromosomal locus). In contrast, loci located close to the *dif* site (presumably in the Ter region where the replication ends) are mostly visualized as one focus (>90% of one-focus cells), indicating that they are segregated immediately prior to cell division. For loci located in between, a linear increase in the proportion of one-focus cells (and therefore a decrease in the proportion of two-foci cells) is observed. Figure 1C shows the percentage of two-foci cells according to cell size for each position. This percentage indicates that the foci duplication process is sequential, proceeding from the origin of replication to the terminus. For each locus, the number of two-foci cells increases with cell size. The curves are remarkably parallel for loci close to *oriC*, and their slope decreases as the distance from *oriC* increases. Very interestingly, for positions close to the *dif* site (2,672-R, 2,957-R and 3,028-R on the right replichore, and 2,784-L and 3,090-L on the left replichore), the proportion of two-foci cells never exceeds 50%, even in larger cells. This finding indicates that the separation of the two copies of these loci occurs concomitantly with cell division in more than 50% of cells.

### The *P. aeruginosa* chromosome shows two distinctive regions surrounding *oriC* and *dif* and is longitudinally organized in between

**Figure 2:**
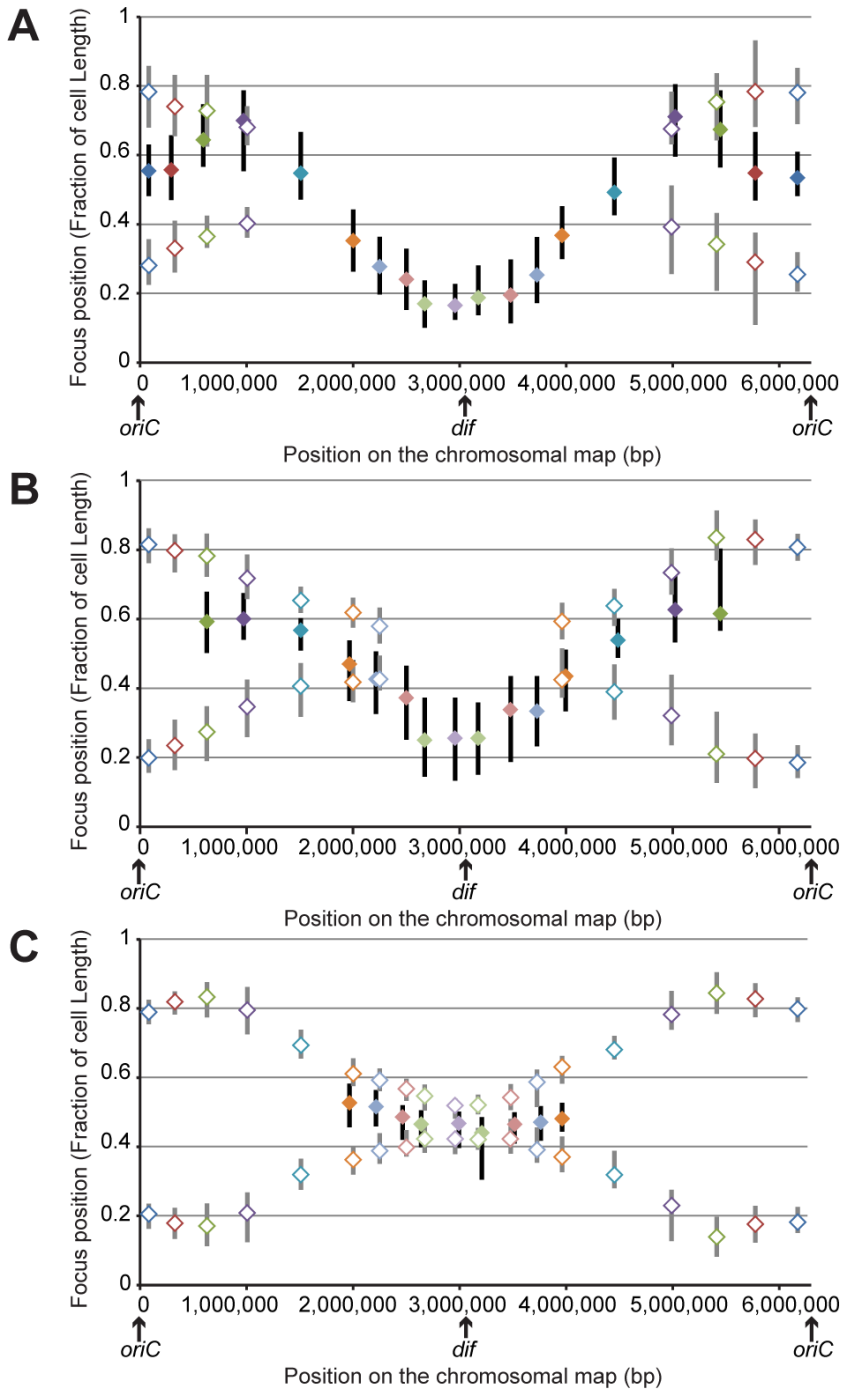
Position of chromosomal loci inside bacterial cells, oriented relative to the new pole of the cell. Positions in cells smaller than 2.8 μm (A), in cells between 2.8 and 3.5 μm (B) and in cells larger than 3.5 μm (C). The *y*-axis represents the relative position of the focus in bacterial cells, 0 being the new pole and 1 the old pole. The markers represent the median values of relative positions, and the vertical bars represent the 25-75th percentiles of the relative positions. The positions of the single focus in one-focus cells are represented with a solid diamond and a black vertical bar, whereas the positions of the two foci in two-foci cells are indicated with an open diamond and a gray vertical bar.

We studied foci localization inside the cells. To orientate bacterial cells, we took advantage of the fact that loci located near *dif* exhibit a striking pattern: in one-focus cells, they are localized close to one pole (closer than the 0.2 relative cell length) in small cells and close to mid-cell in larger cells. In two-foci cells, the 2 foci stay close to the division plane, suggesting that upon division, the pole where the *dif* region is localized is the new pole of the cell. We thus labeled two different loci: one close to *dif*, which we used as a marker of the new pole, and another elsewhere on the chromosome, whose position we were interested in. The data for each locus are presented in Figure S1, and the results are summarized in Figure 2. The relative positions of foci inside bacterial cells are represented for small cells (smaller than 2.8 µm, Figure 2A), medium cells (between 2.8 and 3.5 µm, Figure 2B) and large cells (larger than 3.5 µm, Figure 2C). The proportions of one-focus cells for each cell type are indicated in Table S3. Moreover, Figure S2 presents the same data differently: the relative positions of the foci in cells within 0.2 µm size intervals are presented according to cell size, yielding curves that represent the segregation profiles for each chromosomal locus.

From our results, we inferred the existence of two specific regions in the *P. aeruginosa* chromosome. The first region surrounds *oriC*. Indeed, in large cells (when most of the chromosome is replicated, before cell division), it is clearly observed that the two copies of the three loci closest to *oriC* on each replichore (loci 82-R, 327-R, 628-R and 92-L, 488-L, 851-L) are located at approximately the same position, close to the 0.2/0.8 relative cell length (blue, red and green markers, Figure 2C). Remarkably, eight of the ten *parS* sites found in *P. aeruginosa* are located in between these chromosomal loci (Figure 1A): four of them are very close to *oriC* (between 4 to 15kb from *oriC*), two are found between loci 327-R and 628-R, one is found between loci 92-L and 488-L, and one is found between loci 488-L and 851-L. From these data, we proposed that the three loci closer to *oriC* on each replichore (blue, red and green markers) define a distinctive region that we called the Ori region. Loci in this region segregate sequentially (Figure 1, Figure 2A and 2B) but reach the same final position inside the cell (around the 0.2/0.8 relative cell length).

Loci closest to *oriC* are not the most polarly localized (the red and green markers are farther away from the new pole than the blue marker, Figure 2C), but they are precisely positioned at the 0.2/0.8 relative cell length. This finding is not expected from the position of the loci on the chromosomal map and is not consistent with a strictly longitudinal organization (as in *C. crescentus* chromosomal organization, for instance [28]).

The second region that we discerned in the *P. aeruginosa* chromosome surrounds the *dif* site. Indeed, another striking pattern is observed for the four loci closer to *dif* (positions 2,672-R, 2,957-R, 3,090-L and 2,784-L). As indicated above, they are mostly visualized as a single focus, even in large cells (more than 75% of one-focus cells, Table S3 and Figure 1C). They are located close

to the new pole (below the 0.2 relative cell length) in small cells (Figure 2A); in medium cells, their position is still quite polar (Figure 2B and Figure S2), whereas they are located near mid-cell in large cells (Figure 2C and Figure S2). Because of their polar localization in small cells and the fact that the proportion of cells exhibiting two foci never exceeds 50%, even in large cells, we proposed that these 4 loci belong to another distinctive region that we called the Ter region. Chromosomal locus 3,028-R also belongs to this Ter region, but because it was used to orientate cells, it is not included in Figure 2. Once again, loci in this region segregate sequentially (Figure 1), but their positioning inside the cell is similar.

Chromosomal loci located in between these Ori and Ter regions are spread between the 0.7 and the 0.2 relative cell length when they are visualized as a single focus and between the 0.2/0.8 relative cell length and mid-cell when they are visualized as two foci. This suggests a longitudinal organization of these parts of the *P. aeruginosa* chromosome. To confirm this hypothesis, we measured distances between pairs of loci. We observed that loci located at equivalent distances from *oriC* on different replichores are indeed more frequently colocalized than loci on the same replichore (Figure S3).

Looking more specifically at one-focus cells, most chromosomal loci are relocated close to mid-cell (0.5 relative cell length) before they are resolved into two elements (Figure 2 and Figure S2). This relocation could suggest a sequential repositioning of each chromosomal locus before replication.

### *P. aeruginosa* replisomes are mostly colocalized near mid-cell

To investigate where replication takes place in *P. aeruginosa*, DNA polymerase was visualized in living cells using fusions between replisome components and green fluorescent protein (GFP). This approach has been successfully used in *B. subtilis* [29], *C. crescentus* [30] and *E. coli* [15] to study DNA polymerase localization. Chromosomal genes encoding DnaX (PA1532), HolB (PA2961) and HolA (PA3989) were replaced with genes encoding GFP tagged versions of these proteins (see Text S1 for details). We also engineered a Dronpa-tagged version of DnaX, as Dronpa is a fluorescent protein that has been reported not to cause aberrant foci formation [31]. The results using this DnaX-Dronpa fusion were quite similar to those obtained with DnaX-eGFP (Figure S5).

**Figure 3:**
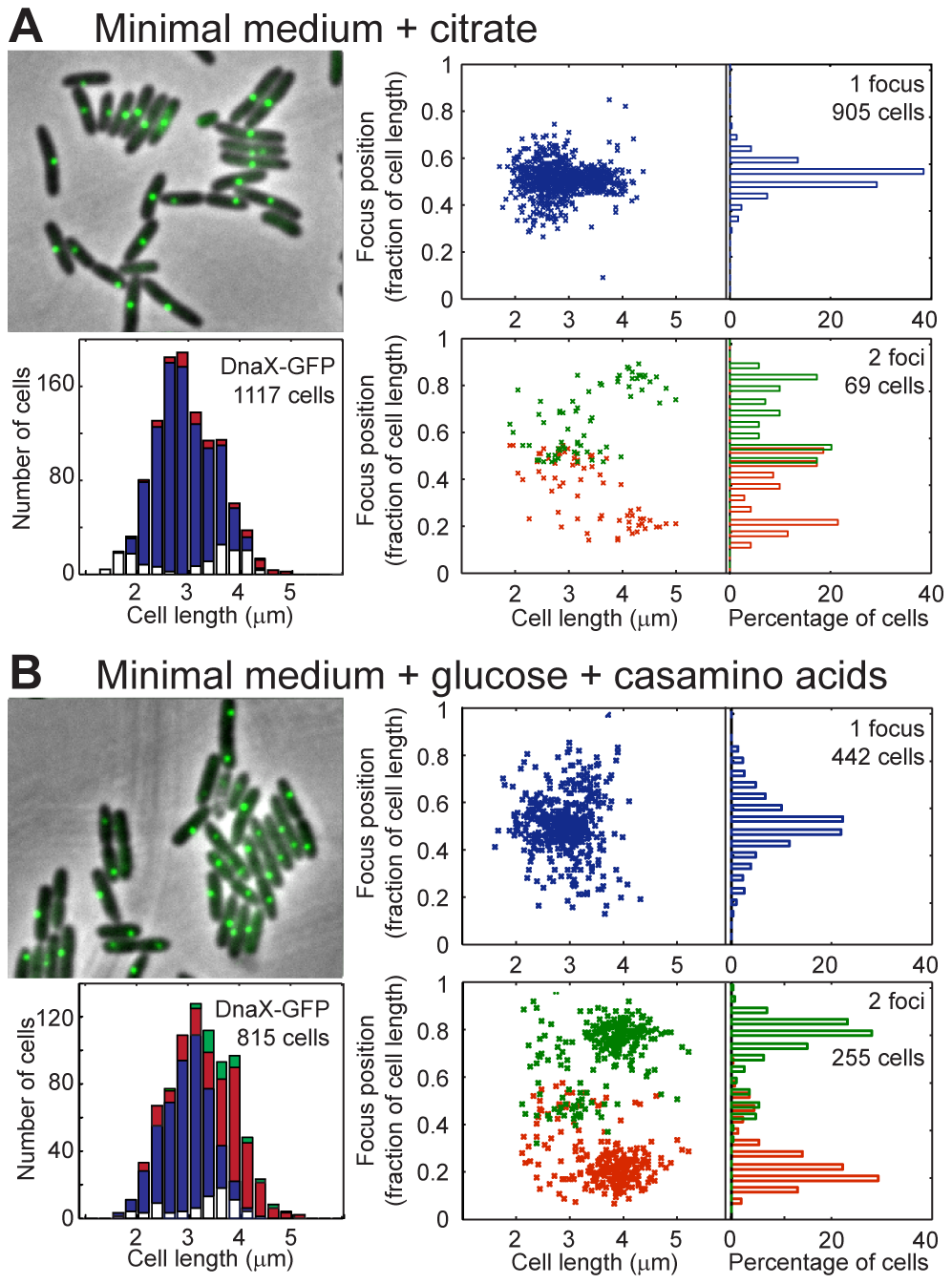
Localization of *P.aeruginosa* DNA polymerase. EGFP-labeled replisome protein DnaX was observed in minimal medium supplemented with citrate (A) or glucose and casamino acids (B). For each panel, the upper left area shows a sample of representative cells; the lower left area presents the amount of cells exhibiting zero (white), one (blue), two (red) or three (green) fluorescent foci according to cell size. The upper right area presents the relative positions of the focus in one-focus cells, and the lower left area presents the relative positions of the foci in two-foci cells.

In minimal medium supplemented with citrate, these GFP fusions were mostly observed as a single spot localized near mid-cell (Figure 3A and Figure S4A). Some cells had no visible focus (about 10%), and although we cannot exclude experimental faults, the highest proportion was observed in smaller cells or larger cells, which is consistent with replication starting immediately upon cell birth and ending before cell division. More than 90% of the cells with foci possessed only one visible focus, indicating that the two replication forks are mostly colocalized. However, a small number of cells presenting two GFP foci were observed for each cell size. In small cells, these foci were found close to mid-cell and relatively close to each other (distance <0.35 of the relative cell length), which suggests that the two forks can separate during the replication process. In the largest cells, however, the two foci were localized far apart, around the 0.2/0.8 relative cell length. These foci coincide with the position of foci close to *oriC*, which could indicate that another round of replication is starting in a few cells, before division is achieved.

Overall, these results show that replication forks are mostly colocalized near mid-cell in *P. aeruginosa*. Therefore, the repositioning of all chromosomal loci close to mid-cell prior to their duplication could indeed be linked to the replication process.

### Global chromosomal organization is conserved in different growth conditions

We next wondered what would happen to chromosomal organization if the growth conditions were changed. In minimal medium supplemented with glucose and casamino acids, 2 to 4 copies of chromosomal loci close to *oriC* were observed (Figure S6), implying that a new round of replication starts before cell division more often in this medium than in citrate medium. The cells are slightly larger in this minimal medium than in minimal medium supplemented with citrate (Figure 3), and the doubling time is similar (approximately 45 minutes, data not shown).

Observing DnaX-eGFP (as well as HolB-eGFP, Figure S4B, and DnaX-Dronpa, Figure S5B) revealed that in one-focus cells, the focus is localized close to mid-cell, as observed in minimal medium supplemented with citrate. The proportion of two-foci cells is higher in MM supplemented with glucose and casamino acids than in MM supplemented with citrate (approximately one third of cells with visible foci presented two foci, Figure 3B). The proportion of two-foci cells increased with cell size: a large majority of cells (approximately 65%) larger than 3.5 µm exhibited two foci. Moreover, a clear difference was observed between the position of the two foci in cells smaller than approximately 3.5 µm, in which the two foci were close together (distance <0.35 of the relative cell length), and the position of the two foci in cells larger than approximately 3.5 µm, where they were located near the one quarter/three quarter position in the cells. These results suggest that in cells smaller than approximately 3.5 µm, a replication round is finishing near mid-cell, most often with the two forks colocalized. In contrast, in cells larger than approximately 3.5 µm, another round of replication started on the two duplicated chromosomes, close to the mid-cell of the future daughter cells. The two replication forks that reproduce the same chromosome are also mostly colocalized.

In contrast to loci close to *oriC* (which are present in two to four copies), intermediate chromosomal loci (1,812-L and 1,509-R) are observed as only one or two foci (Figure S6), which might suggest that cell division occurs before the replication forks reach these positions. Loci located near *dif* (3,090-L and 2,499-R) show the same localization pattern in minimal medium supplemented with glucose and casamino acids as in minimal medium supplemented with citrate: they are localized near the cell pole in small cells and near mid-cell in larger cells. A single focus near mid-cell can be observed in large cells despite the fact that the chromosome is fully replicated (as can be inferred from visualizing replisome proteins). When a locus in the Ori region and a locus in the Ter region are visualized in the same cells, the proportion of cells exhibiting four foci of the Ori locus and one focus of the Ter locus is twice the proportion of cells exhibiting four foci of the Ori locus and two focus of the Ter locus (data not shown). This finding suggests an extensive colocalization step following replication for the Ter region.

Together, these results suggest that in minimal medium supplemented with glucose and casamino acids, the *P. aeruginosa* chromosome is organized similarly to how it is organized in minimal medium supplemented with citrate, except that two chromosomes are being replicated instead of one (see discussion).

### Disruption of the ParABS system has a major impact on chromosomal organization

**Figure 4:**
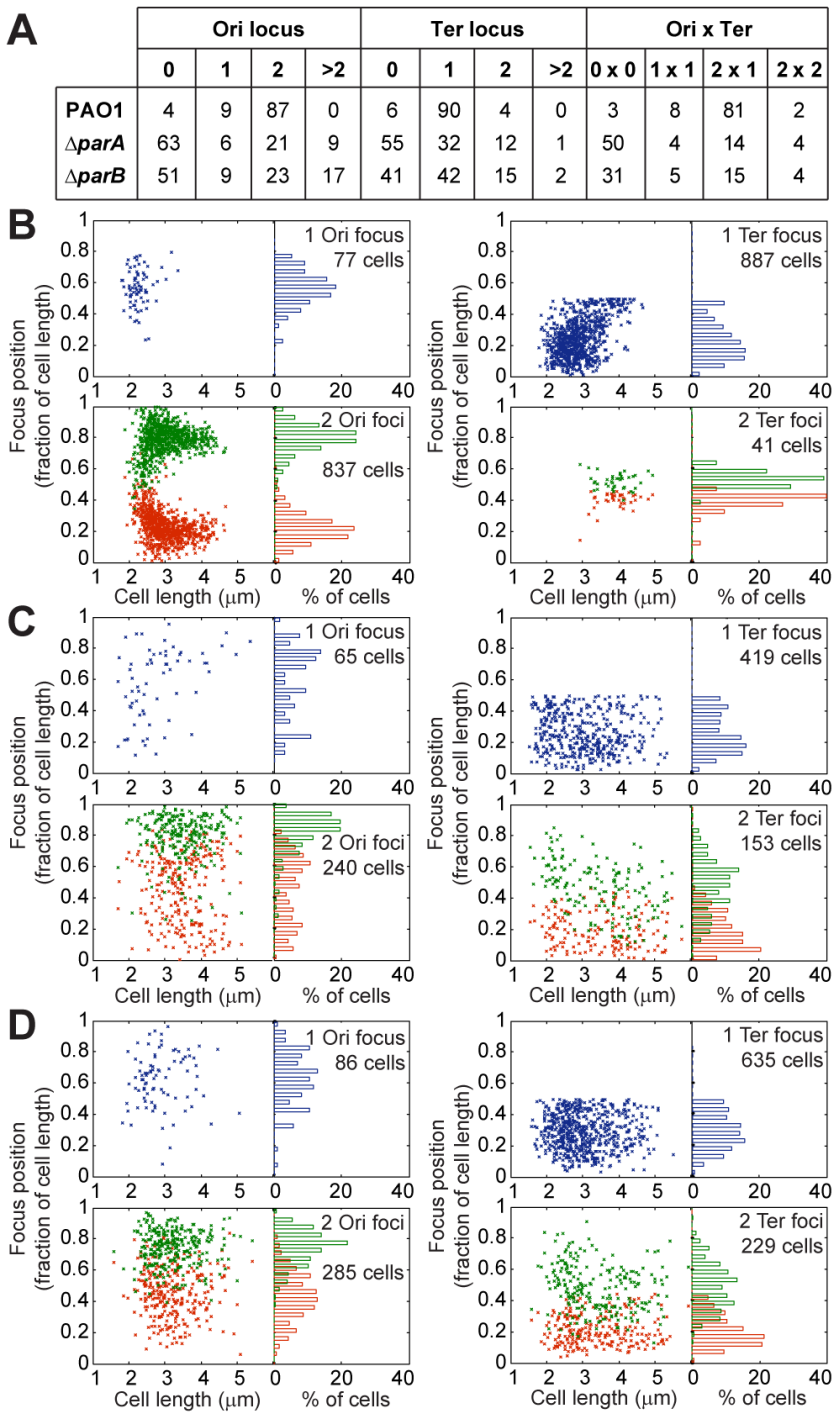
Extensive chromosomal disorganization in mutants of the ParABS system. (A) Percentages of the population presenting a given number of foci corresponding to the Ter locus or the Ori locus. Positioning of chromosomal loci located in the Ori region (left panels) and in the Ter region (right panels) in a wild type PAO1 strain (B), the Δ*parA* mutant (C) and the Δ*parB* mutant (D) grown in minimal medium supplemented with citrate. The position of the foci in cells containing 1 (upper panels) or 2 (bottom panels) foci are presented. Ori locus: 82-R in (A), (B) and (C). Ter loci: 2,957-R in (A), 3,090-L in (B) and (C).

As a first step to study molecular mechanisms involved in *P. aeruginosa* chromosomal organization and segregation, we disrupted the ParABS system by deleting either the *parA* gene or the *parB* gene. Consistent with previously reported work [24], the Δ*parA* mutant showed an increase in its doubling time (approximately 100 minutes in minimal medium supplemented with citrate, as opposed to approximately 50 minutes for the wild-type strain, data not shown). In the genetic context we used, the Δ*parB* mutant showed the same growth defect, in contrast to previous observations reporting a growth rate only 5 to 10% lower than that of the wild type strain [25]. Both mutants produced more than 20% anucleated cells when grown in minimal medium supplemented with citrate (data not shown), suggesting a major defect in chromosomal segregation. To further investigate the impact of these mutations on chromosomal organization, we analyzed the positioning of chromosomal loci located in the Ter and Ori regions during growth in minimal medium supplemented with citrate. The results are shown in Figure 4. Consistent with the large number of anucleated cells, more than half the cells exhibited no visible foci for both the Ter and the Ori loci. Additionally, the proportion of cells containing a given number of foci in the Δ*par* mutants is strikingly different from the wild type strain (Figure 4A). For instance, only 15% of the cells have one visible Ter focus and two visible Ori foci in these mutants, compared to more than 80% of the wild type cells.

Moreover, the localization of these foci inside the cells also completely changed (Figure 4B–4D). A strong segregation defect is observed for the Ori locus in both the Δ*parA* and the Δ*parB* mutants: the two copies of this locus are not segregated to the 0.2/0.8 relative cell length. Furthermore, the typical localization pattern of the Ter locus is completely lost in the Δ*parA* and Δ*parB* mutants, which indicates that the whole chromosomal organization is altered when the ParABS system is disrupted.

## Discussion

**Figure 5:**
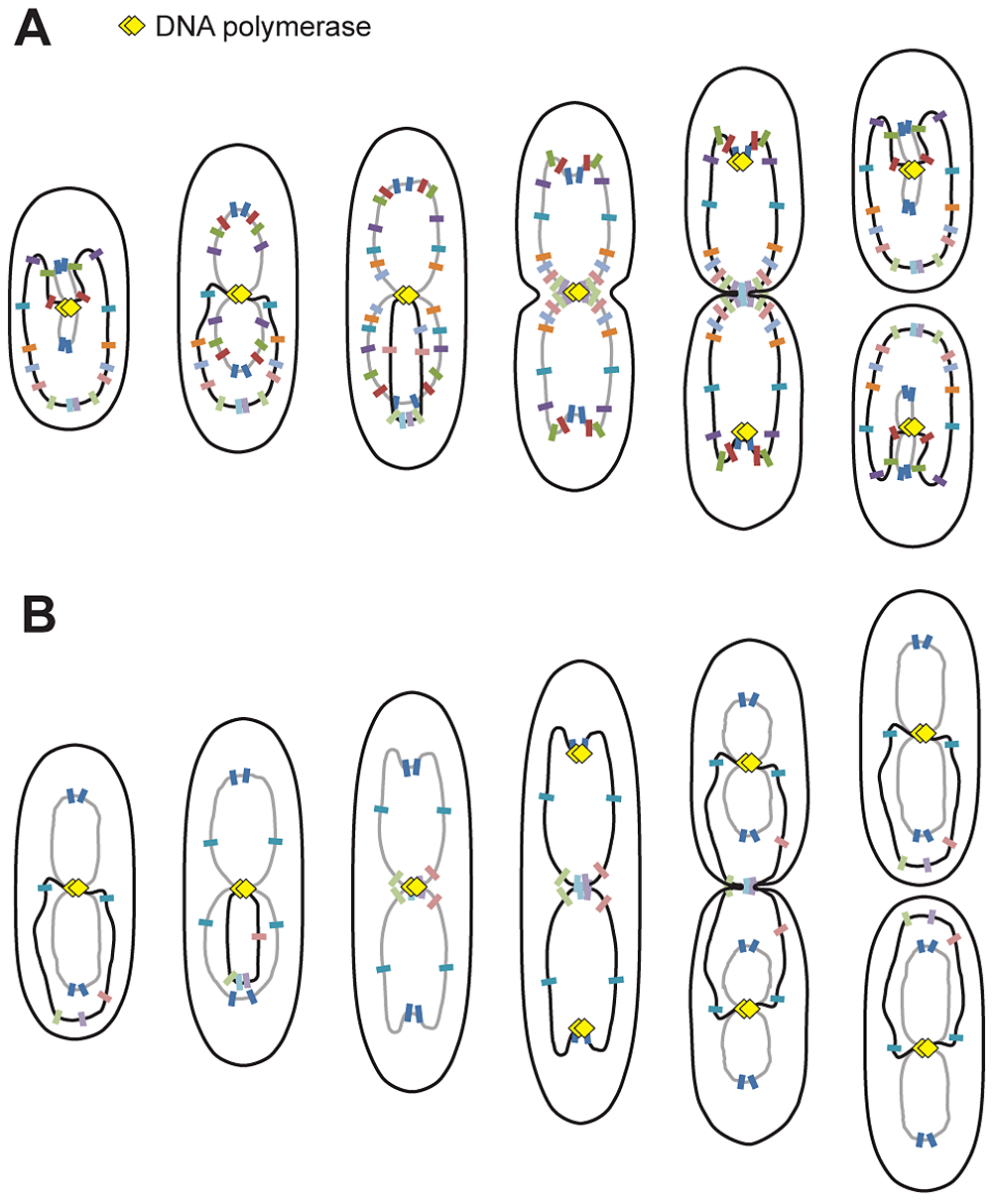
Proposed model for *P. aeruginosa* chromosomal organization. Organization in minimal medium supplemented with citrate (A) or glucose and casamino acids (B). Black lines represent fully replicated chromosomes, whereas grey lines represent partially replicated chromosomes. Colored markers represent chromosomal loci, and yellow diamonds represent replisomes.

In this study, we showed that the *P. aeruginosa* chromosome is longitudinally organized between 2 large regions in which loci are localized at a similar position inside the cell upon segregation, one surrounding *oriC* (approximately 1.4 Mbp) and the other surrounding *dif* (approximately 800 kb). Segregation occurs sequentially along the Ori/Ter axis and follows replication, which occurs near mid-cell (Figure 5). Segregation and positioning of the *P. aeruginosa* chromosome rely on a functional ParABS system.

### *P. aeruginosa*'s chromosome is longitudinally organized between 2 distinctive regions

The longitudinal organization of the *P. aeruginosa* chromosome is similar to chromosomal organization in *C. crescentus*. However, in *C. crescentus*, a linear correspondence exists between the position of any given chromosomal locus and its position inside the bacterial cell [28]; however, this is not always the case in *P. aeruginosa* for loci that belong to the Ori or Ter regions. Indeed, loci belonging to these regions are localized around the same position before and after segregation: loci from the Ori region are near the 0.2/0.8 relative cell length in large cells and loci from the Ter region are below the 0.2 relative cell length in small cells. Large domains have only been described thus far in *E. coli* [11], [32], which exhibits a transversal organization of its chromosome: *oriC* and the Ter region are localized near mid-cell, while the replichores are localized in different halves of the cell [27], [33]. *P. aeruginosa* is thus the first bacterium in which a unique combination of properties could coexist: longitudinal positioning and the presence of large distinctive regions. A systematic study of the positioning of chromosomal loci in *B. subtilis* or *V. cholera* has yet to be published.

### The 1.4 Mb region surrounding *oriC* could constitute a specific region

In *P. aeruginosa*, the three loci closest to *oriC* on each replichore (encompassing approximately 1.4 Mbp around *oriC*) appear to be localized at the same position upon segregation (See model, Figure 5). Interestingly, this region includes the eight *parS* sites located around *oriC* [34], suggesting a putative role of the ParABS system in organizing this specific region, in addition to its global role in the segregation of the *P. aeruginosa* chromosome. In *C. crescentus*, the most polar region of the chromosome contains two *parS* sites (which are located 8 kb away from *oriC* in the wild type chromosomal configuration). In *P. aeruginosa*, the whole Ori region is localized near the 0.2/0.8 relative cell length position. The two loci closest to *oriC* (82-R and 92-L) are precisely positioned, whereas loci 327-R, 628-R, 488-L and 851-L can be localized slightly more towards the cell pole (Figure 2C). Even if *parS* sites are dispersed in this Ori region, four of them are located between +4 kb and +15 kb from *oriC*, which could account for the precise positioning of the loci closest to *oriC*. Deletion or displacement of *parS* sites will be necessary to determine their role in the positioning and existence of the Ori region as we defined it.

It is worth noting that in *C. crescentus*, sporulating *B. subtilis* and *V. cholerae*, *oriC* is more polarly localized than what we observed in *P. aeruginosa*. Proteins anchoring *oriC* to the cell pole via the ParABS system have been identified in these bacteria [35]–[38] and are not found in *P. aeruginosa*. The FimV protein, a homolog of HubP that is responsible for pole anchorage in *V. cholerae*, lacks the specific domain responsible for interacting with the ParA protein [38]. It is thus very unlikely that it plays a role in positioning the Ori region of *P. aeruginosa* at the 0.2/0.8 relative cell length position. The mechanisms responsible for this precise localization have yet to be identified.

In *B. subtilis*, ParB (Spo0J) binds to *parS* and recruits the SMC complex, which plays a major role in organizing the origin region and promotes chromosomal segregation [21], [22]. Two condensins have been identified in *P. aeruginosa*, the SMC complex and the MksB complex, which could also be involved in chromosomal segregation [23]. The role of these proteins in organizing *P. aeruginosa*'s chromosome has yet to be carefully investigated, as well as their interplay with the ParABS system. We might imagine that the strong detrimental effect of the deletion of *parB* is linked to the inability of the condensins to be recruited to the Ori region, as observed in *B. subtilis* [21], [22]. However, the deletion of *parA* in *P. aeruginosa* is also very detrimental to the cell, in contrast to what is observed in *B. subtilis*.

Considering the major impact on chromosomal organization and segregation of the impairment of the ParABS system in *P. aeruginosa*, the development of specific tools will be required to more specifically analyze the role of ParA and ParB in organizing the Ori region, as well as their interplay with Smc and MksB during chromosome segregation.

### Properties of the Ter region in *P. aeruginosa*

Another striking observation in *P. aeruginosa* is that loci located in the approximately 800 kb surrounding the *dif* site show a characteristic localization during the cell cycle. We arbitrarily define the Ter region as containing these loci, which are positioned below the 0.2 relative cell length in small cells and are sequentially repositioned near mid-cell prior to their duplication. Interestingly, loci in the Ter region are mostly visualized as one single focus, even when they are obviously already duplicated. This distinctive feature is clearly observed when cells are grown in minimal medium supplemented with glucose and casamino acids. In these conditions, large cells possess 2 complete chromosomes that are being replicated (see model, Figure 5), and nevertheless, loci close to *dif* are frequently visualized as 1 focus. In most cases, the separation of these Ter loci is concomitant with cell division and might require septum formation. Further analyses will be required to elucidate the nature of this Ter region and to identify processes that specifically control the segregation of loci belonging to that region.

A specific domain surrounding the *dif* site was characterized in *E. coli*. This domain, called the Ter domain, was first identified using a cellular biological approach [11], [39]. Loci in this domain also present a long colocalization step at mid-cell following replication [14], [32], [39]. A specific factor involved in this colocalization step, called MatP, has been identified [40], [41]. MatP recognizes a specific sequence, *matS*, that is repeated 23 times in the Ter region of *E. coli* and interacts with the divisome. No MatP homolog is found in *P. aeruginosa*, and preliminary bioinformatic analysis did not identify a sequence characteristic of the Ter region of *P. aeruginosa*. Therefore, processes responsible for specifying the Ter region of the *P. aeruginosa* chromosome may rely on different molecular mechanisms. It is interesting to note that even if domains have not been characterized in *C. crescentus* and *V. cholerae*, one locus located near *dif* in these bacteria also remains colocalized at mid-cell after replication [42], [43].

### Replication occurs at mid-cell in *P. aeruginosa*

This work also addressed the localization of replisomes near mid-cell in *P. aeruginosa*. This localization is reminiscent of that described in *B. subtilis* [29]. In *C. crescentus*, replisomes are also often colocalized, but they move from one pole to mid-cell during replication [30] following chromosomal organization. In *E. coli*, the two replication forks appear to follow chromosomal arms as they separate into the two halves of the cell [14], [15]. Based on our analysis of the localization of replisome proteins, it appears that replication lasts for most of the cell cycle when *P. aeruginosa* is grown in minimal medium supplemented with citrate (see model, Figure 5). In newborn cells, loci closest to *oriC* are being replicated and are found near mid-cell together with DNA polymerase. As cells grow, replisomes stay near mid-cell, where loci are successively relocated prior to their replication. This relocation may result from the replication process, which could be responsible for pulling DNA towards mid-cell.

Overall, this study allowed us to give a precise description of chromosomal organization in *P. aeruginosa*. This organization is original, combining large distinctive regions and a longitudinal positioning. These results pave the way for additional work that will lead to the understanding of the mechanisms responsible for such organization, which will contribute to our appreciation of the wide diversity of mechanisms used by bacteria, even when the most fundamental processes are concerned. This work might also allow us to identify drugs that would interfere with fundamental processes such as chromosomal replication and segregation in this important pathogen.

## Materials and Methods

### Plasmids and strains

*P. aeruginosa* strain PAO1 was provided by Arne Rietsch (Case Western Reserve University). This PAO1 strain does not present the inversion described for the sequenced PAO1-UW subclone resulting from homologous recombination between the *rrnA* and *rrnB* loci, which are orientated in opposite directions and separated by 2.2 Mbp [4]. This explains the discrepancies between PA numbers and positions on the chromosomal map, as gene annotation was done in the inversion containing strain. Chromosomal loci were called according to their position from *oriC* on each replichore. Details of plasmid and strain construction are provided in Text S1, Table S1 and Table S2.

### Fluorescence microscopy analysis

Strains were grown overnight in LB, diluted 300 times in minimal medium A supplemented with either 0.25% citrate or 0.12% casamino acids and 0.5% glucose (when looking at chromosomal tags, 0.5 mM IPTG was added to growth medium) until they reach an OD600 comprised between 0.05 and 0.1. Cells were then spread on agarose pads and immediately observed using a Leica DM6000 microscope, a coolsnap HQ CCD camera (Roper) and Metamorph software. Image analysis was performed using the MATLAB-based software MicrobeTracker Suite [44].

## Supporting Information

Figure S1 Data related to Figure 2 and Figure S2, representing the positions of chromosomal loci relative to the new pole of the cell. A chromosomal locus near *dif* was used to orientate cells (Strains used are indicated in bold in Table S2). For each locus, the upper panel represents the position of the focus in one focus cells, and the lower panel represents the position of the 2 foci in 2 foci cells (in red the focus proximal to the new pole of the cell). The x-axis in the left part of the panel represents cell size, whereas the x-axis of the right part of the panel represents the number of cells. The y-axis represents the relative position of the focus, 0 being the new pole and 1 the old pole of the cell. More than 800 cells were analyzed for each locus. Experiments were performed 2 to 4 times independently, and a representative set of experiments is shown here.
(TIF)

Figure S2 Positions of chromosomal loci relative to the new pole of the cell, according to the cell size. For each chromosomal locus, median value(s) of the relative position(s) of the focus/foci are indicated by a colored horizontal bar for cells of a certain size (cell size categories have been defined every 0.2 µm). The y-axis represents the relative position of the focus in bacterial cells, 0 being the new pole and 1 the old pole. The x-axis represents cell length. When the proportion of one-focus cells in the cell size category was higher than 50%, the median value of the position of the single focus in one- focus cells are represented, otherwise the median values of the positions of the two foci in two-foci cells are represented. The black horizontal bars represent the position of DnaX-CFP relative to the new pole of the cell. Similarly to what was described for chromosomal loci, cells are oriented using a chromosomal locus located near *dif*.
(TIF)

Figure S3 Loci on different replichores are more frequently colocalized than loci on the same replichore. (A) Schematic representation of the different combinations of chromosomal loci analyzed here. Colored arrows points to chromosomal loci whose interfocal distances are represented in (B). (B) Interfocal distance between 2 chromosomal loci located on different replichores (blue and red) or on the same replichore (green, purple or orange). X-axis represents the interfocal distance between 2 loci, and the y-axis represents the proportion of cells in the size category. (C) Mean interfocal distance for each loci combination.
(TIF)

Figure S4 Localization of *P. aeruginosa* DNA polymerase using a HolB-eGFP fusion. EGFP-labeled replisome protein HolB was observed in minimal medium supplemented with citrate (A) or glucose and casamino acids (B). For each panel, the upper left part shows a sample of representative cells; the lower left part represent the amount of cells presenting zero (white), one (blue) or two (red) fluorescent foci, according to cell size. The upper right part represents the relative positions of the 1 focus in 1 focus cells, and the lower left part represents the relative positions of the 2 foci in 2 foci cells.
(TIF)

Figure S5 Localization of *P. aeruginosa* DNA polymerase using a DnaX-Dronpa fusion. Dronpa-labeled replisome protein DnaX was observed in minimal medium supplemented with citrate (A) or glucose and casamino acids (B). For each panel, the upper left part shows a sample of representative cells; the lower left part represent the amount of cells presenting zero (white), one (blue) or two (red) fluorescent foci, according to cell size. The upper right part represents the relative positions of the one focus in one-focus cells, and the lower left part represents the relative positions of the two foci in two-foci cells.
(TIF)

Figure S6 Positions of chromosomal loci when cells are grown in minimal medium supplemented with glucose and casamino acids. The same representation as in Figure S1 is used. The proportion of cells with one, two, three or four foci is indicated for each chromosomal locus. For loci 92-L, 82-R, 3,090-R and 2,499-R, cells were oriented using another chromosomal locus near *dif* which allows identifying the new pole of the cells, whereas for loci 1,812-L and 1,509-R cells were randomly oriented.
(TIF)

Table S1 Plasmids and oligonucleotides used in this study.
(DOCX)

Table S2 Strains carrying chromosomal tags used in this study. The strains indicated in bold are the one used to position chromosomal loci relative to the new pole of the cells.
(DOCX)

Table S3 Percentage of one-focus cells for each chromosomal locus for each cell type in oriented cells (related to Figure 2).
(DOCX)

Text S1 Supporting Materials and Methods.
(DOCX)

## References

[1] Silby MW, Winstanley C, Godfrey SA, Levy SB, Jackson RW (2011) *Pseudomonas* genomes: diverse and adaptable. FEMS Microbiol Rev 35: 652–680. 21361996 10.1111/j.1574-6976.2011.00269.x

[2] Govan JR, Deretic V (1996) Microbial pathogenesis in cystic fibrosis: mucoid *Pseudomonas aeruginosa* and *Burkholderia cepacia* . Microbiol Rev 60: 539–574. 8840786 10.1128/mr.60.3.539-574.1996 PMC239456

[3] Lister PD, Wolter DJ, Hanson ND (2009) Antibacterial-resistant *Pseudomonas aeruginosa*: clinical impact and complex regulation of chromosomally encoded resistance mechanisms. Clin Microbiol Rev 22: 582–610. 19822890 10.1128/CMR.00040-09 PMC2772362

[4] Stover CK, Pham XQ, Erwin AL, Mizoguchi SD, Warrener P, et al (2000) Complete genome sequence of *Pseudomonas aeruginosa* PAO1, an opportunistic pathogen. Nature 406: 959–964. 10984043 10.1038/35023079

[5] Yee TW, Smith DW (1990) *Pseudomonas* chromosomal replication origins: a bacterial class distinct from *Escherichia coli*-type origins. Proc Natl Acad Sci U S A 87: 1278–1282. 2106132 10.1073/pnas.87.4.1278 PMC53457

[6] Jiang Y, Yao S, Helinski D, Toukdarian A (2006) Functional analysis of two putative chromosomal replication origins from *Pseudomonas aeruginosa* . Plasmid 55: 194–200. 16376988 10.1016/j.plasmid.2005.11.001

[7] de Vries R (2010) DNA condensation in bacteria: Interplay between macromolecular crowding and nucleoid proteins. Biochimie 92: 1715–1721. 20615449 10.1016/j.biochi.2010.06.024

[8] Thanbichler M, Shapiro L (2006) Chromosome organization and segregation in bacteria. J Struct Biol 156: 292–303. 16860572 10.1016/j.jsb.2006.05.007

[9] Toro E, Shapiro L (2010) Bacterial chromosome organization and segregation. Cold Spring Harb Perspect Biol 2: a000349. 20182613 10.1101/cshperspect.a000349 PMC2828278

[10] Possoz C, Junier I, Espeli O (2012) Bacterial chromosome segregation. Front Biosci 17: 1020–1034. 10.2741/3971 22201788

[11] Niki H, Yamaichi Y, Hiraga S (2000) Dynamic organization of chromosomal DNA in Escherichia coli. Genes Dev 14: 212–223. 10652275 PMC316355

[12] Valens M, Penaud S, Rossignol M, Cornet F, Boccard F (2004) Macrodomain organization of the *Escherichia coli* chromosome. EMBO J 23: 4330–4341. 15470498 10.1038/sj.emboj.7600434 PMC524398

[13] Lemon KP, Grossman AD (2001) The extrusion-capture model for chromosome partitioning in bacteria. Genes Dev 15: 2031–2041. 11511534 10.1101/gad.913301

[14] Bates D, Kleckner N (2005) Chromosome and replisome dynamics in *E. coli*: loss of sister cohesion triggers global chromosome movement and mediates chromosome segregation. Cell 121: 899–911. 15960977 10.1016/j.cell.2005.04.013 PMC2973560

[15] Reyes-Lamothe R, Possoz C, Danilova O, Sherratt DJ (2008) Independent positioning and action of *Escherichia coli* replisomes in live cells. Cell 133: 90–102. 18394992 10.1016/j.cell.2008.01.044 PMC2288635

[16] Jun S, Wright A (2010) Entropy as the driver of chromosome segregation. Nat Rev Microbiol 8: 600–607. 20634810 10.1038/nrmicro2391 PMC3148256

[17] Fogel MA, Waldor MK (2006) A dynamic, mitotic-like mechanism for bacterial chromosome segregation. Genes Dev 20: 3269–3282. 17158745 10.1101/gad.1496506 PMC1686604

[18] Toro E, Hong SH, McAdams HH, Shapiro L (2008) *Caulobacter* requires a dedicated mechanism to initiate chromosome segregation. Proc Natl Acad Sci U S A 105: 15435–15440. 18824683 10.1073/pnas.0807448105 PMC2563096

[19] Lee PS, Lin DC, Moriya S, Grossman AD (2003) Effects of the chromosome partitioning protein Spo0J (ParB) on oriC positioning and replication initiation in *Bacillus subtilis* . J Bacteriol 185: 1326–1337. 12562803 10.1128/JB.185.4.1326-1337.2003 PMC142880

[20] Minnen A, Attaiech L, Thon M, Gruber S, Veening JW (2011) SMC is recruited to oriC by ParB and promotes chromosome segregation in *Streptococcus pneumoniae* . Mol Microbiol 81: 676–688. 21651626 10.1111/j.1365-2958.2011.07722.x

[21] Sullivan NL, Marquis KA, Rudner DZ (2009) Recruitment of SMC by ParB-parS organizes the origin region and promotes efficient chromosome segregation. Cell 137: 697–707. 19450517 10.1016/j.cell.2009.04.044 PMC2892783

[22] Gruber S, Errington J (2009) Recruitment of condensin to replication origin regions by ParB/SpoOJ promotes chromosome segregation in *B. subtilis* . Cell 137: 685–696. 19450516 10.1016/j.cell.2009.02.035

[23] Petrushenko ZM, She W, Rybenkov VV (2011) A new family of bacterial condensins. Mol Microbiol 81: 881–896. 21752107 10.1111/j.1365-2958.2011.07763.x PMC3179180

[24] Lasocki K, Bartosik AA, Mierzejewska J, Thomas CM, Jagura-Burdzy G (2007) Deletion of the parA (soj) homologue in *Pseudomonas aeruginosa* causes ParB instability and affects growth rate, chromosome segregation, and motility. J Bacteriol 189: 5762–5772. 17545287 10.1128/JB.00371-07 PMC1951838

[25] Bartosik AA, Mierzejewska J, Thomas CM, Jagura-Burdzy G (2009) ParB deficiency in *Pseudomonas aeruginosa* destabilizes the partner protein ParA and affects a variety of physiological parameters. Microbiology 155: 1080–1092. 19332810 10.1099/mic.0.024661-0 PMC2895232

[26] Lau IF, Filipe SR, Soballe B, Okstad OA, Barre FX, et al (2003) Spatial and temporal organization of replicating *Escherichia coli* chromosomes. Mol Microbiol 49: 731–743. 12864855 10.1046/j.1365-2958.2003.03640.x

[27] Nielsen HJ, Ottesen JR, Youngren B, Austin SJ, Hansen FG (2006) The *Escherichia coli* chromosome is organized with the left and right chromosome arms in separate cell halves. Mol Microbiol 62: 331–338. 17020576 10.1111/j.1365-2958.2006.05346.x

[28] Viollier PH, Thanbichler M, McGrath PT, West L, Meewan M, et al (2004) Rapid and sequential movement of individual chromosomal loci to specific subcellular locations during bacterial DNA replication. Proc Natl Acad Sci U S A 101: 9257–9262. 15178755 10.1073/pnas.0402606101 PMC438963

[29] Lemon KP, Grossman AD (1998) Localization of bacterial DNA polymerase: evidence for a factory model of replication. Science 282: 1516–1519. 9822387 10.1126/science.282.5393.1516

[30] Jensen RB, Wang SC, Shapiro L (2001) A moving DNA replication factory in *Caulobacter crescentus* . EMBO J 20: 4952–4963. 11532959 10.1093/emboj/20.17.4952 PMC125615

[31] Landgraf D, Okumus B, Chien P, Baker TA, Paulsson J (2012) Segregation of molecules at cell division reveals native protein localization. Nat Methods 9: 480–482. 22484850 10.1038/nmeth.1955 PMC3779060

[32] Espeli O, Mercier R, Boccard F (2008) DNA dynamics vary according to macrodomain topography in the *E. coli* chromosome. Mol Microbiol 68: 1418–1427. 18410497 10.1111/j.1365-2958.2008.06239.x

[33] Wang X, Possoz C, Sherratt DJ (2005) Dancing around the divisome: asymmetric chromosome segregation in *Escherichia coli* . Genes Dev 19: 2367–2377. 16204186 10.1101/gad.345305 PMC1240045

[34] Bartosik AA, Lasocki K, Mierzejewska J, Thomas CM, Jagura-Burdzy G (2004) ParB of *Pseudomonas aeruginosa*: interactions with its partner ParA and its target parS and specific effects on bacterial growth. J Bacteriol 186: 6983–6998. 15466051 10.1128/JB.186.20.6983-6998.2004 PMC522188

[35] Ben-Yehuda S, Rudner DZ, Losick R (2003) RacA, a bacterial protein that anchors chromosomes to the cell poles. Science 299: 532–536. 12493822 10.1126/science.1079914

[36] Bowman GR, Comolli LR, Zhu J, Eckart M, Koenig M, et al (2008) A polymeric protein anchors the chromosomal origin/ParB complex at a bacterial cell pole. Cell 134: 945–955. 18805088 10.1016/j.cell.2008.07.015 PMC2745220

[37] Ebersbach G, Briegel A, Jensen GJ, Jacobs-Wagner C (2008) A self-associating protein critical for chromosome attachment, division, and polar organization in *caulobacter* . Cell 134: 956–968. 18805089 10.1016/j.cell.2008.07.016 PMC2614312

[38] Yamaichi Y, Bruckner R, Ringgaard S, Moll A, Cameron DE, et al (2012) A multidomain hub anchors the chromosome segregation and chemotactic machinery to the bacterial pole. Genes Dev 26: 2348–2360. 23070816 10.1101/gad.199869.112 PMC3475806

[39] Li Y, Youngren B, Sergueev K, Austin S (2003) Segregation of the *Escherichia coli* chromosome terminus. Mol Microbiol 50: 825–834. 14617144 10.1046/j.1365-2958.2003.03746.x

[40] Mercier R, Petit MA, Schbath S, Robin S, El Karoui M, et al (2008) The MatP/matS site-specific system organizes the terminus region of the *E. coli* chromosome into a macrodomain. Cell 135: 475–485. 18984159 10.1016/j.cell.2008.08.031

[41] Espeli O, Borne R, Dupaigne P, Thiel A, Gigant E, et al (2012) A MatP-divisome interaction coordinates chromosome segregation with cell division in *E. coli* . EMBO J 31: 3198–3211. 22580828 10.1038/emboj.2012.128 PMC3400007

[42] Jensen RB (2006) Coordination between chromosome replication, segregation, and cell division in *Caulobacter crescentus* . J Bacteriol 188: 2244–2253. 16513754 10.1128/JB.188.6.2244-2253.2006 PMC1428140

[43] Srivastava P, Fekete RA, Chattoraj DK (2006) Segregation of the replication terminus of the two *Vibrio* cholerae chromosomes. J Bacteriol 188: 1060–1070. 16428410 10.1128/JB.188.3.1060-1070.2006 PMC1347332

[44] Sliusarenko O, Heinritz J, Emonet T, Jacobs-Wagner C (2011) High-throughput, subpixel precision analysis of bacterial morphogenesis and intracellular spatio-temporal dynamics. Mol Microbiol 80: 612–627. 21414037 10.1111/j.1365-2958.2011.07579.x PMC3090749

