# Supplementary figures and images for: Chromosomal Organization and Segregation in Pseudomonas aeruginosa
Source: PLoS Genet. 2013 May 2;9(5):e1003492. doi: 10.1371/journal.pgen.1003492 (PMC3642087; doi:10.1371/journal.pgen.1003492)

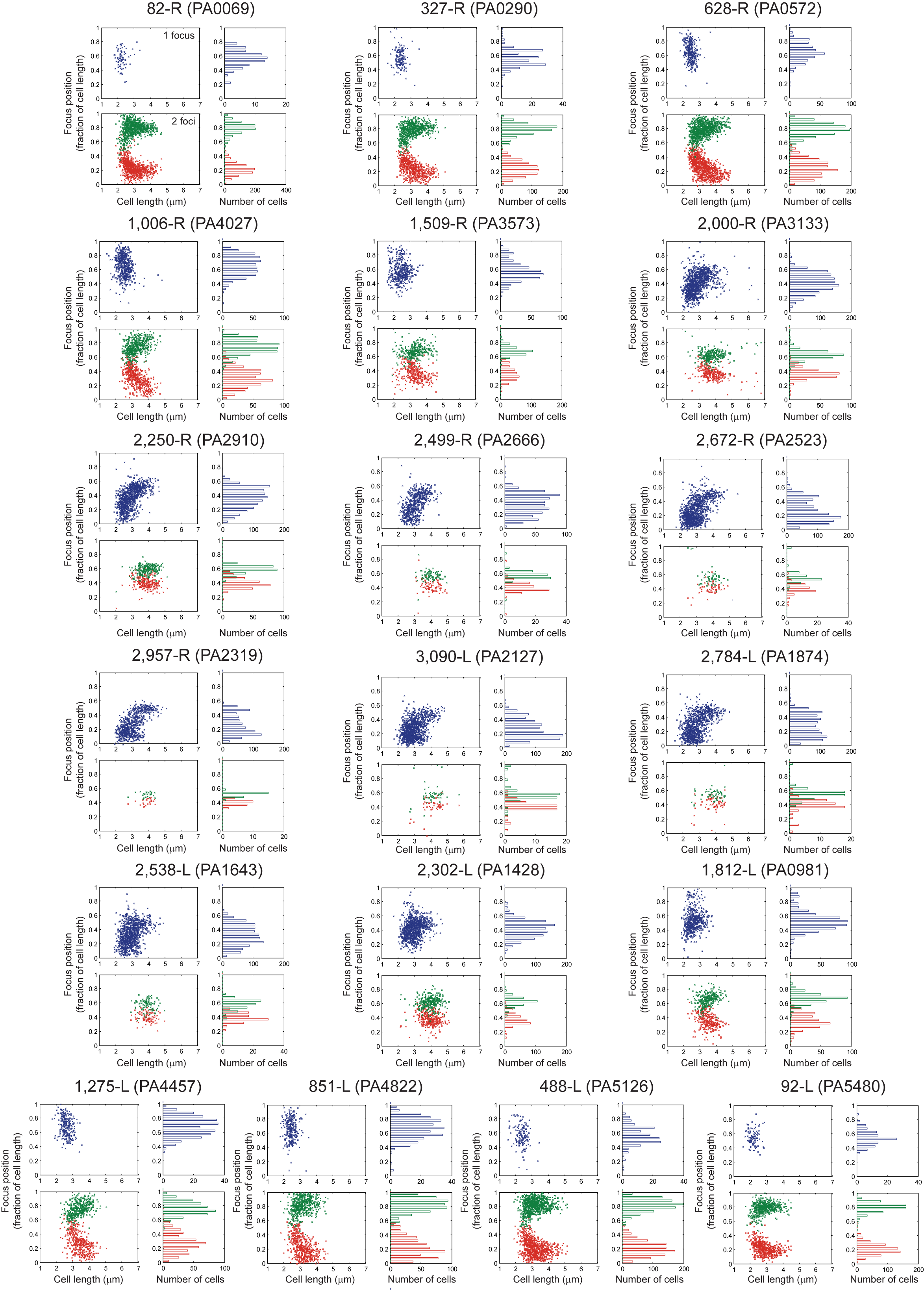

Supplement: Figure S1 — Data related to Figure 2 and Figure S2, representing the positions of chromosomal loci relative to the new pole of the cell. A chromosomal locus near dif was used to orientate cells (Strains used are indicated in bold in Table S2). For each locus, the upper panel represents the position of the focus in one focus cells, and the lower panel represents the position of the 2 foci in 2 foci cells (in red the focus proximal to the new pole of the cell). The x-axis in the left part of the panel represents cell size, whereas the x-axis of the right part of the panel represents the number of cells. The y-axis represents the relative position of the focus, 0 being the new pole and 1 the old pole of the cell. More than 800 cells were analyzed for each locus. Experiments were performed 2 to 4 times independently, and a representative set of experiments is shown here. (TIF) [file pgen.1003492.s001.tif]

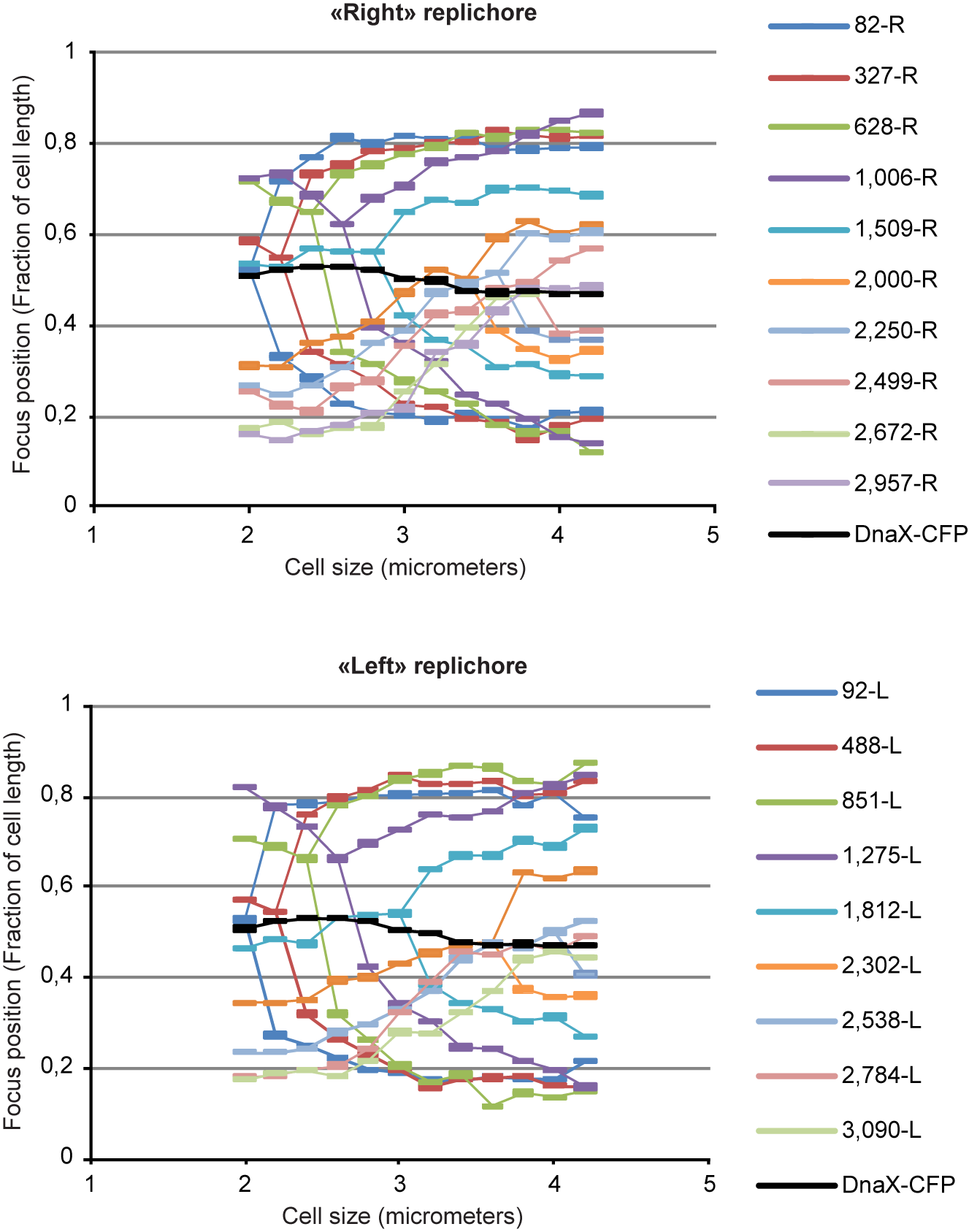

Supplement: Figure S2 — Positions of chromosomal loci relative to the new pole of the cell, according to the cell size. For each chromosomal locus, median value(s) of the relative position(s) of the focus/foci are indicated by a colored horizontal bar for cells of a certain size (cell size categories have been defined every 0.2 µm). The y-axis represents the relative position of the focus in bacterial cells, 0 being the new pole and 1 the old pole. The x-axis represents cell length. When the proportion of one-focus cells in the cell size category was higher than 50%, the median value of the position of the single focus in one- focus cells are represented, otherwise the median values of the positions of the two foci in two-foci cells are represented. The black horizontal bars represent the position of DnaX-CFP relative to the new pole of the cell. Similarly to what was described for chromosomal loci, cells are oriented using a chromosomal locus located near dif. (TIF) [file pgen.1003492.s002.tif]

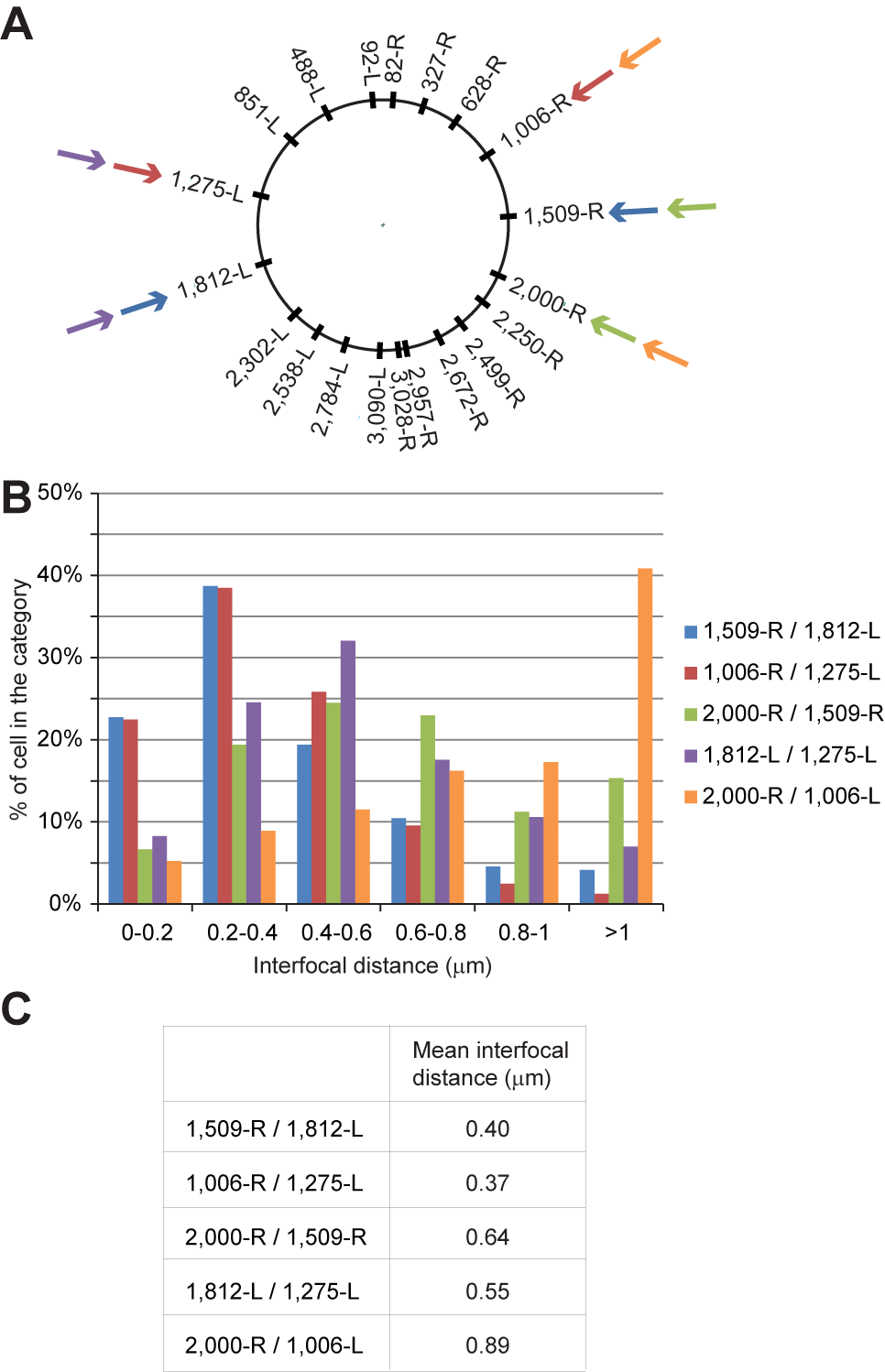

Supplement: Figure S3 — Loci on different replichores are more frequently colocalized than loci on the same replichore. (A) Schematic representation of the different combinations of chromosomal loci analyzed here. Colored arrows points to chromosomal loci whose interfocal distances are represented in (B). (B) Interfocal distance between 2 chromosomal loci located on different replichores (blue and red) or on the same replichore (green, purple or orange). X-axis represents the interfocal distance between 2 loci, and the y-axis represents the proportion of cells in the size category. (C) Mean interfocal distance for each loci combination. (TIF) [file pgen.1003492.s003.tif]

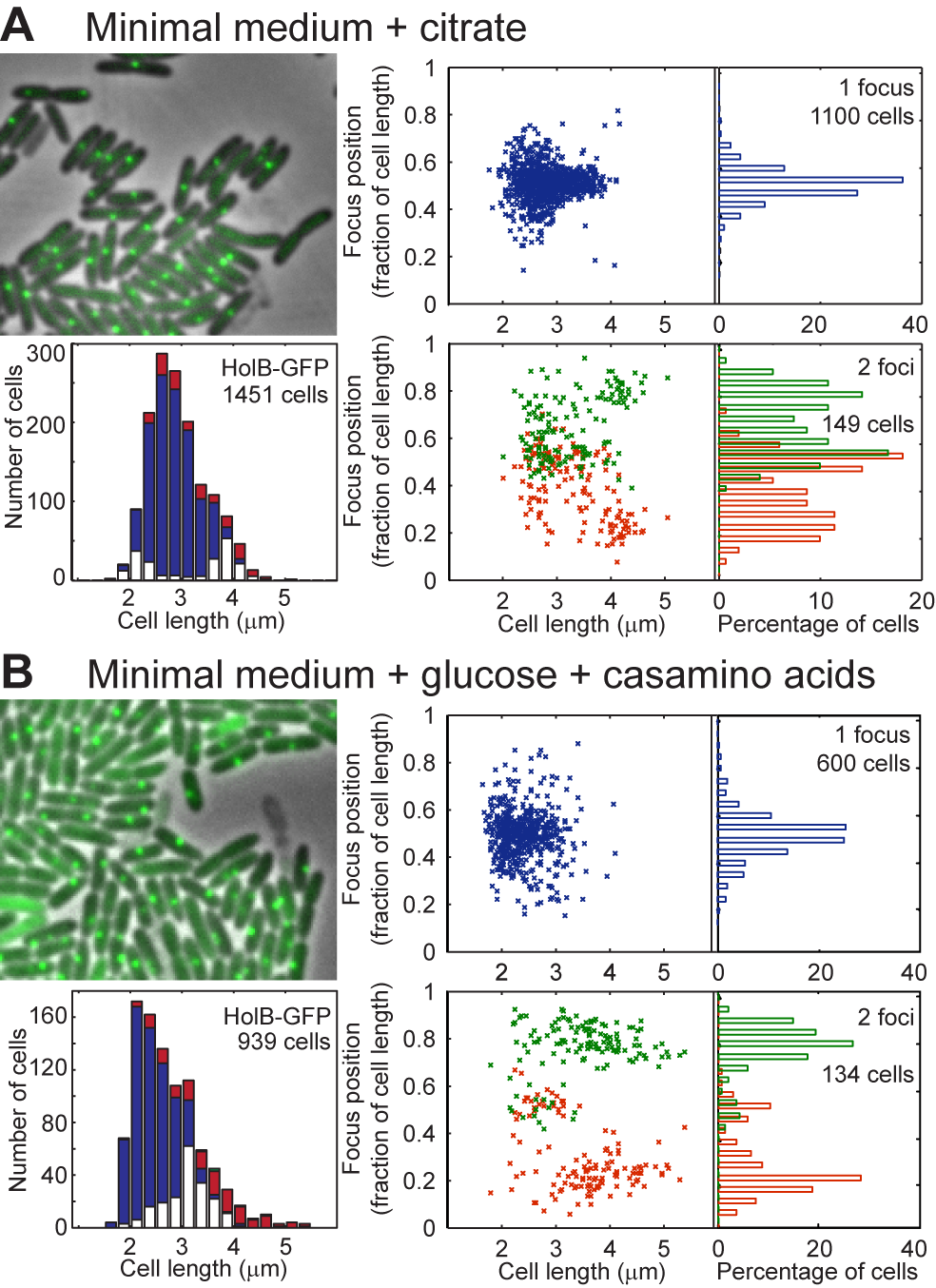

Supplement: Figure S4 — Localization of P. aeruginosa DNA polymerase using a HolB-eGFP fusion. EGFP-labeled replisome protein HolB was observed in minimal medium supplemented with citrate (A) or glucose and casamino acids (B). For each panel, the upper left part shows a sample of representative cells; the lower left part represent the amount of cells presenting zero (white), one (blue) or two (red) fluorescent foci, according to cell size. The upper right part represents the relative positions of the 1 focus in 1 focus cells, and the lower left part represents the relative positions of the 2 foci in 2 foci cells. (TIF) [file pgen.1003492.s004.tif]

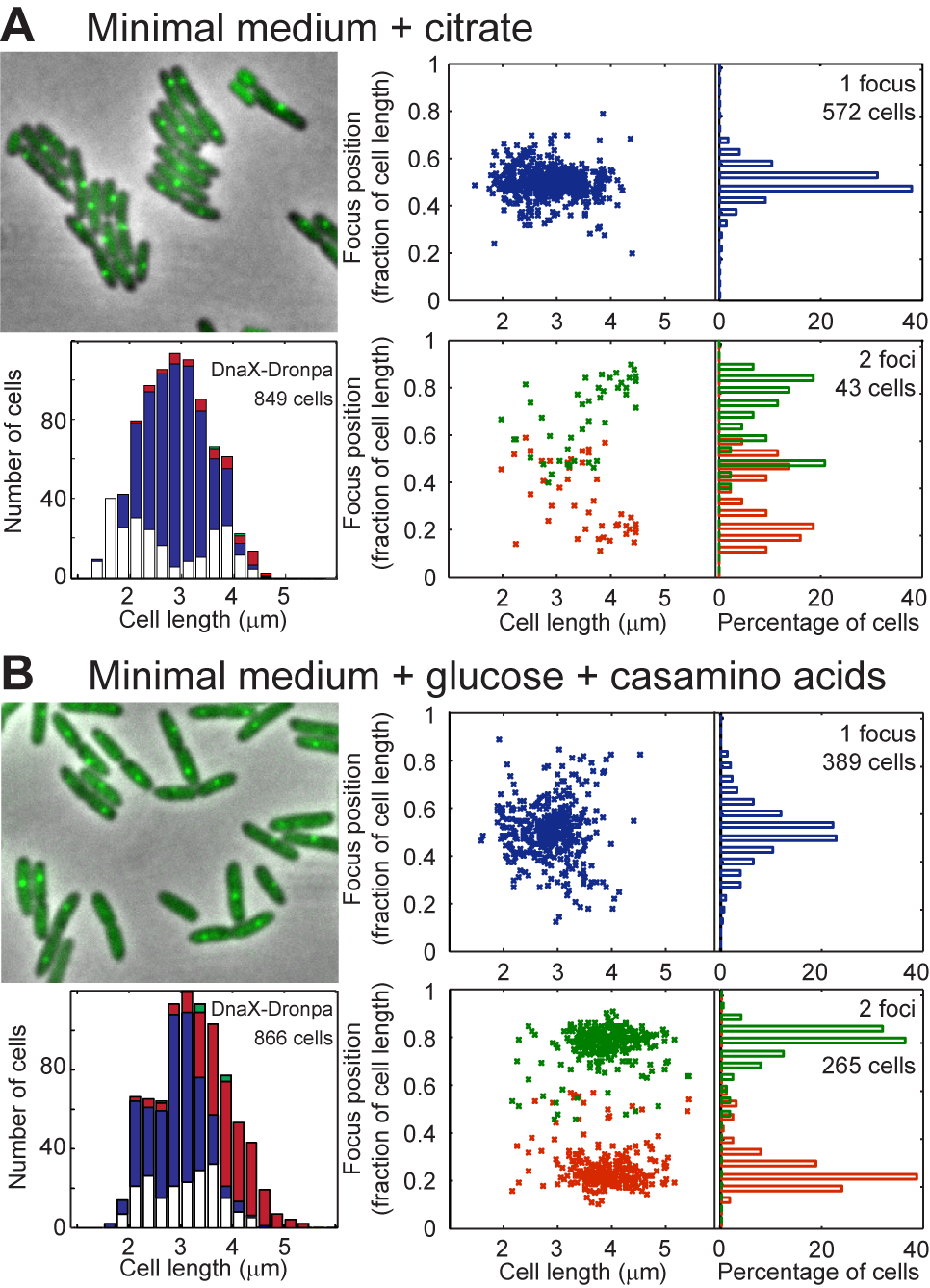

Supplement: Figure S5 — Localization of P. aeruginosa DNA polymerase using a DnaX-Dronpa fusion. Dronpa-labeled replisome protein DnaX was observed in minimal medium supplemented with citrate (A) or glucose and casamino acids (B). For each panel, the upper left part shows a sample of representative cells; the lower left part represent the amount of cells presenting zero (white), one (blue) or two (red) fluorescent foci, according to cell size. The upper right part represents the relative positions of the one focus in one-focus cells, and the lower left part represents the relative positions of the two foci in two-foci cells. (TIF) [file pgen.1003492.s005.tif]

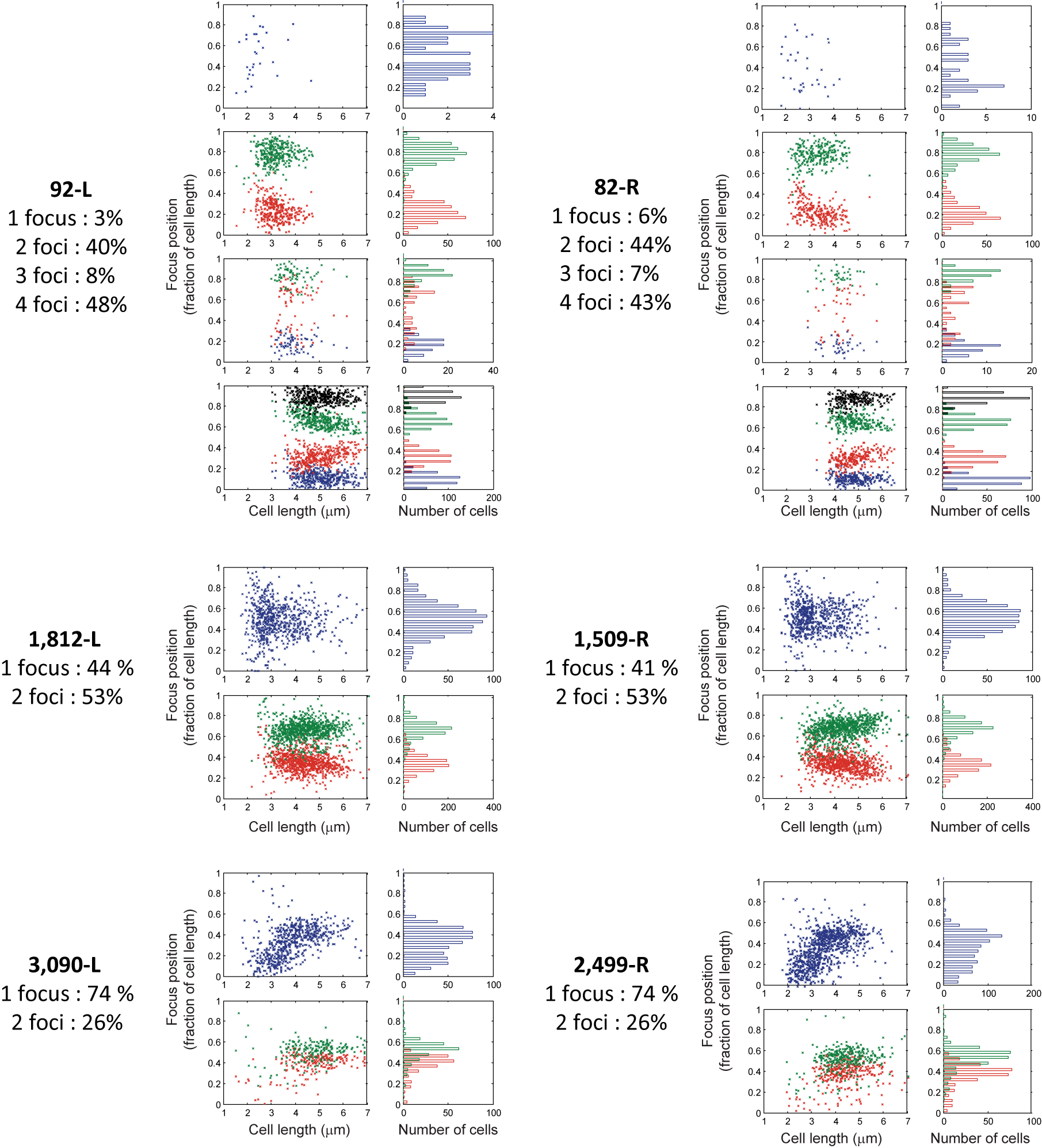

Supplement: Figure S6 — Positions of chromosomal loci when cells are grown in minimal medium supplemented with glucose and casamino acids. The same representation as in Figure S1 is used. The proportion of cells with one, two, three or four foci is indicated for each chromosomal locus. For loci 92-L, 82-R, 3,090-R and 2,499-R, cells were oriented using another chromosomal locus near dif which allows identifying the new pole of the cells, whereas for loci 1,812-L and 1,509-R cells were randomly oriented. (TIF) [file pgen.1003492.s006.tif]
