# Supplementary material for: Chromosomal Organization and Segregation in Pseudomonas aeruginosa
Source: PLoS Genet. 2013 May 2;9(5):e1003492. doi: 10.1371/journal.pgen.1003492 (PMC3642087; doi:10.1371/journal.pgen.1003492)
Supplement: Table S1 — Plasmids and oligonucleotides used in this study. (DOCX) [file pgen.1003492.s007.docx]

**Table S1 :** Plasmids and oligonucleotides used in this study.

| **Plasmid** | **Oligos used to amplify and clone chromosomal regions** | **Position of the chromosomal tag** | **Distance from *oriC* - (name of the chromosomal tag)** |
| --- | --- | --- | --- |
| pP30D-FRT-parST1-0069 pP30D-FRT-tetO-0069 | ATAAGCTTAAGCGCATTCTCGAACCG ATGGTACCCTGCTTCCGACGTTTCCG | 82,283 | 82,283 (82-R) |
| pP30D-FRT-parST1-0290 pP30D-FRT-tetO-0290 | ATAAGCTTCGTTTCCGTTTTGTCGAC ATGGTACCTCGTTGAAACACTGAGCG | 327,196 | 327,196 (327-R) |
| pP30D-FRT-parST1-0572 pP30D-FRT-tetO-0572 | ATAAGCTTCGGTGGCGAGGGATGGGT ATGGTACCCACTTATCAGGCACTTCC | 628,184 | 628,184 (628-R) |
| pP30D-FRT-parST1-4027 pP30D-FRT-tetO-4027 | ATAAGCTTCCAGGAGCAGGACCAGGG ATGGTACCCCGCTGGAACCGCAATGC | 1,006,073 | 1,006,073 (1,006-R) |
| pP30D-FRT-parST1-3573 pP30D-FRT-tetO-3573 | ATAAGCTTCTCGGCAGGTAGAAATCG ATGGTACCACGGGTTTTTTCAGAATC | 1,509,300 | 1,509,300 (1,509-R) |
| pP30D-FRT-parST1-3133 | ATAAGCTTTATTATGGATGAAATATT ATGGTACCGGATTCCTACGAAGGTTT | 2,000,736 | 2,000,736 (2,000-R) |
| pP30D-FRT-parST1-2910 pP30D-FRT-tetO-2910 | ATAAGCTTTTCGGCGCAGGAATGTCG ATGGTACCCCGGCTCGCTCCCGCCCA | 2,250,069 | 2,250,069 (2,250-R) |
| pP30D-FRT-parST1-2666 pP30D-FRT-tetO-2666 | ATAAGCTTCTGGATCTGGCAACAACT ATGGTACCTTTCCAGCAGGGCCAGTT | 2,499,532 | 2,499,532 (2,499-R) |
| pP30D-FRT-parST1-2523 pP30D-FRT-tetO-2523 | ATAAGCTTGTTCCGCTCCTCGTCTGC ATGGTACCGTTCGCCCCTATATAAAG | 2,672,028 | 2,672,028 (2,672-R) |
| pP30D-FRT-parST1-2319 pP30D-FRT-tetO-2319 | ATAAGCTTCGGGGCTGGAAATCCTTA ATGGTACCGCGACGCTGCCCAACCCT | 2,957,361 | 2,957,361 (2,957-R) |
| pP30D-FRT-parST1-2258 pP30D-FRT-tetO-2258 | ATAAGCTTCGGAAACGCTGCTGATGT ATGGTACCTACTTGAGAAACTACTTT | 3,028,240 | 3,028,240 (3,028-R) |
| pP30D-FRT-parST1-2127 pP30D-FRT-tetO-2127 | ATAAGCTTCTCCAGGTCGATGAACAG ATGGTACCGCCGCCGCTCAGAAGTCC | 3,173,821 | 3,090,583 (3,090-L) |
| pP30D-FRT-parST1-1874 pP30D-FRT-tetO-2874 | ATAAGCTTCCGTAGATCAGGCCGAAC ATGGTACCTGTAATCCAGAAATAGCA | 3,479,449 | 2,784,955 (2,784-L) |
| pP30D-FRT-parST1-1643 pP30D-FRT-tetO-1643 | ATAAGCTTCAAGGAAGACCGCGTCGA ATGGTACCGGAGGGGACGCCGATTAT | 3,726,010 | 2,538,394 (2,538-L) |
| pP30D-FRT-parST1-1428 pP30D-FRT-tetO-1428 | ATAAGCTTGCGGAACAACGGTCGGTC ATGGTACCCTTCCCATGCTTTTCCCG | 3,961,614 | 2,302,790 (2,302-L) |
| pP30D-FRT-parST1-0981 pP30D-FRT-tetO-0981 | ATAAGCTTTCGTTTGGCTGAGGGCTA ATGGTACCCTTCATCGCTTCCCCAAA | 4,451,691 | 1,812,713 (1,812-L) |
| pP30D-FRT-parST1-4457 pP30D-FRT-tetO-4457 | ATAAGCTTGATGTTGTCGAAAATCGC ATGGTACCTGTTCAACGAATGTGGCG | 4,989,294 | 1,275,110 (1,275-L) |
| pP30D-FRT-parST1-4822 pP30D-FRT-tetO-4822 | ATAAGCTTGGCTGGTCCTGGCCGCCT ATGGTACCGTCACCGCCCTGGCCTAT | 5,412,656 | 851,748 (851-L) |
| pP30D-FRT-parST1-5126 pP30D-FRT-tetO-5126 | ATAAGCTTTCCGCAGCACAGCCCCAG ATGGTACCGATTCCCCAGCCTGCCTG | 5,775,422 | 488,982 (488-L) |
| pP30D-FRT-parST1-5480 pP30D-FRT-tetO-5480 | ATAAGCTTTAAGTGACTGCCCC ATGGTACCGGCGGGTTCCAGGG | 6,172,140 | 92,264 (92-L) |
