# Supplementary material for: Chromosomal Organization and Segregation in Pseudomonas aeruginosa
Source: PLoS Genet. 2013 May 2;9(5):e1003492. doi: 10.1371/journal.pgen.1003492 (PMC3642087; doi:10.1371/journal.pgen.1003492)
Supplement: Table S2 — Strains carrying chromosomal tags used in this study. The strains indicated in bold are the one used to position chromosomal loci relative to the new pole of the cells. (DOCX) [file pgen.1003492.s008.docx]

**Table S2 :** Strains carrying chromosomal tags used in this study. The strains indicated in bold are the one used to position chromosomal loci relative to the new pole of the cells.

| **Strain name** | **Strain description** |
| --- | --- |
| **IVGB118** | **PAO1 parST1-PA5480(92-L) tetO-PA2319(2,957-R) + pPSV35Ap-TetR-Cfp-yGfp-ParBT1** |
| IVGB119 | PAO1 parST1-PA3573(1,509-R) tetO-PA4457(1,275-L) + pPSV35Ap-TetR-Cfp-yGfp-ParBT1 |
| IVGB120 | PAO1 parST1-PA2127(3,090-L) tetO-PA2319(2,957-R) + pPSV35Ap-TetR-Cfp-yGfp-ParBT1 |
| IVGB121 | PAO1 parST1-PA2127(3,090-L) tetO-PA2666(2,499-R) + pPSV35Ap-TetR-Cfp-yGfp-ParBT1 |
| **IVGB123** | **PAO1 parST1-PA2319(2,957-R) tetO-PA0069(82-R) + pPSV35Ap-TetR-Cfp-yGfp-ParBT1** |
| IVGB125 | PAO1 parST1-PA3573(1,509-R) tetO-PA0981(1,812-L) + pPSV35Ap-TetR-Cfp-yGfp-ParBT1 |
| IVGB126 | PAO1 parST1-PA2666(2,499-R) tetO-PA0572(628-R) + pPSV35Ap-TetR-Cfp-yGfp-ParBT1 |
| IVGB127 | PAO1 parST1-PA0981(1,812-L) tetO-PA4457(1,275-L) + pPSV35Ap-TetR-Cfp-yGfp-ParBT1 |
| IVGB128 | PAO1 parST1-PA5126(488-L) tetO-PA0069(82-R) + pPSV35Ap-TetR-Cfp-yGfp-ParBT1 |
| IVGB168 | PAO1 parST1-PA0572(628-R) tetO-PA0069(82-R) + pPSV35Ap-TetR-Cfp-yGfp-ParBT1 |
| IVGB169 | PAO1 parST1-PA0572(628-R) tetO-PA0290(327-R) + pPSV35Ap-TetR-Cfp-yGfp-ParBT1 |
| IVGB170 | PAO1 parST1-PA0572(628-R) tetO-PA4027(1,006-R) + pPSV35Ap-TetR-Cfp-yGfp-ParBT1 |
| IVGB171 | PAO1 parST1-PA5126(488-L) tetO-PA4822(851-L) + pPSV35Ap-TetR-Cfp-yGfp-ParBT1 |
| IVGB172 | PAO1 parST1-PA5480(92-L) tetO-PA5126(488-L) + pPSV35Ap-TetR-Cfp-yGfp-ParBT1 |
| IVGB173 | PAO1 parST1-PA5480(92-L) tetO-PA4822(851-L) + pPSV35Ap-TetR-Cfp-yGfp-ParBT1 |
| IVGB174 | PAO1 parST1-PA4457(1,275-L) tetO-PA4822(851-L) + pPSV35Ap-TetR-Cfp-yGfp-ParBT1 |
| **IVGB175** | **PAO1 parST1-PA2127(3,090-L) tetO-PA1874(2,784-L) + pPSV35Ap-TetR-Cfp-yGfp-ParBT1** |
| **IVGB176** | **PAO1 parST1-PA2127(3,090-L) tetO-PA1643(2,538-L) + pPSV35Ap-TetR-Cfp-yGfp-ParBT1** |
| **IVGB177** | **PAO1 parST1-PA2127(3,090-L) tetO-PA1428(2,302-L) + pPSV35Ap-TetR-Cfp-yGfp-ParBT1** |
| **IVGB178** | **PAO1 parST1-PA2127(3,090-L) tetO-PA2258(3,028-R) + pPSV35Ap-TetR-Cfp-yGfp-ParBT1** |
| **IVGB179** | **PAO1 parST1-PA2319(2,957-R) tetO-PA2523(2,672-R) + pPSV35Ap-TetR-Cfp-yGfp-ParBT1** |
| **IVGB180** | **PAO1 parST1-PA2319(2,957-R) tetO-PA2666(2,499-R) + pPSV35Ap-TetR-Cfp-yGfp-ParBT1** |
| **IVGB181** | **PAO1 parST1-PA2319(2,957-R) tetO-PA2910(2,250-R) + pPSV35Ap-TetR-Cfp-yGfp-ParBT1** |
| **IVGB182** | **PAO1 parST1-PA2319(2,957-R) tetO-PA2258(3,028-R) + pPSV35Ap-TetR-Cfp-yGfp-ParBT1** |
| IVGB247 | PAO1 parST1-PA1428(2,302-L) tetO-PA0981(1,812-L) + pPSV35Ap-TetR-Cfp-yGfp-ParBT1 |
| IVGB248 | PAO1 parST1-PA1428(2,302-L) tetO-PA4457(1,275-L) + pPSV35Ap-TetR-Cfp-yGfp-ParBT1 |
| IVGB249 | PAO1 parST1-PA1428(2,302-L) tetO-PA4822(851-L) + pPSV35Ap-TetR-Cfp-yGfp-ParBT1 |
| IVGB250 | PAO1 parST1-PA1428(2,302-L) tetO-PA1643(2,538-L) + pPSV35Ap-TetR-Cfp-yGfp-ParBT1 |
| IVGB251 | PAO1 parST1-PA1428(2,302-L) tetO-PA1874(2,784-L) + pPSV35Ap-TetR-Cfp-yGfp-ParBT1 |
| IVGB252 | PAO1 parST1-PA3133(2,000-R) tetO-PA3573(1,509-R) + pPSV35Ap-TetR-Cfp-yGfp-ParBT1 |
| IVGB253 | PAO1 parST1-PA3133(2,000-R) tetO-PA4027(1,006-R) + pPSV35Ap-TetR-Cfp-yGfp-ParBT1 |
| IVGB254 | PAO1 parST1-PA3133(2,000-R) tetO-PA2910(2,250-R) + pPSV35Ap-TetR-Cfp-yGfp-ParBT1 |
| IVGB255 | PAO1 parST1-PA3133(2,000-R) tetO-PA2666(2,499-R) + pPSV35Ap-TetR-Cfp-yGfp-ParBT1 |
| **IVGB256** | **PAO1 parST1-PA3133(2,000-R) tetO-PA2523(2,672-R) + pPSV35Ap-TetR-Cfp-yGfp-ParBT1** |
| IVGB257 | PAO1 parST1-PA1874(2,784-L) tetO-PA1643(2,538-L) + pPSV35Ap-TetR-Cfp-yGfp-ParBT1 |
| IVGB258 | PAO1 parST1-PA1874(2,784-L) tetO-PA1428(2,302-L) + pPSV35Ap-TetR-Cfp-yGfp-ParBT1 |
| IVGB287 | PAO1 parST1-PA0572(628-R) tetO-PA0981(1,812-L) + pPSV35Ap-TetR-Cfp-yGfp-ParBT1 |
| IVGB288 | PAO1 parST1-PA4457(1,275-L) tetO-PA4027(1,006-R) + pPSV35Ap-TetR-Cfp-yGfp-ParBT1 |
| IVGB289 | PAO1 parST1-PA5480(92-L) tetO-PA4457(1,275-L) + pPSV35Ap-TetR-Cfp-yGfp-ParBT1 |
| IVGB290 | PAO1 parST1-PA5480(92-L) tetO-PA0981(1,812-L) + pPSV35Ap-TetR-Cfp-yGfp-ParBT1 |
| IVGB291 | PAO1 parST1-PA5480(92-L) tetO-PA1428(2,302-L) + pPSV35Ap-TetR-Cfp-yGfp-ParBT1 |
| IVGB292 | PAO1 parST1-PA0069(82-R) tetO-PA0290(327-R) + pPSV35Ap-TetR-Cfp-yGfp-ParBT1 |
| IVGB293 | PAO1 parST1-PA0069(82-R) tetO-PA4027(1,006-R) + pPSV35Ap-TetR-Cfp-yGfp-ParBT1 |
| IVGB294 | PAO1 parST1-PA0069(82-R) tetO-PA3573(1,509-R) + pPSV35Ap-TetR-Cfp-yGfp-ParBT1 |
| **IVGB296** | **PAO1 parST1-PA2319(2,957-R) tetO-PA3573(1,509-R) + pPSV35Ap-TetR-Cfp-yGfp-ParBT1** |
| **IVGB297** | **PAO1 parST1-PA2319(2,957-R) tetO-PA4027(1,006-R) + pPSV35Ap-TetR-Cfp-yGfp-ParBT1** |
| **IVGB298** | **PAO1 parST1-PA2319(2,957-R) tetO-PA0572(628-R) + pPSV35Ap-TetR-Cfp-yGfp-ParBT1** |
| **IVGB299** | **PAO1 parST1-PA2319(2,957-R) tetO-PA0290(327-R) + pPSV35Ap-TetR-Cfp-yGfp-ParBT1** |
| **IVGB301** | **PAO1 parST1-PA2127(3,090-L) tetO-PA0981(1,812-L) + pPSV35Ap-TetR-Cfp-yGfp-ParBT1** |
| **IVGB302** | **PAO1 parST1-PA2127(3,090-L) tetO-PA4457(1,275-L) + pPSV35Ap-TetR-Cfp-yGfp-ParBT1** |
| **IVGB303** | **PAO1 parST1-PA2127(3,090-L) tetO-PA4822(851-L) + pPSV35Ap-TetR-Cfp-yGfp-ParBT1** |
| **IVGB304** | **PAO1 parST1-PA2127(3,090-L) tetO-PA5126(488-L) + pPSV35Ap-TetR-Cfp-yGfp-ParBT1** |
| IVGB305 | PAO1 parST1-PA2523(2,672-R) tetO-PA2910(2,250-R) + pPSV35Ap-TetR-Cfp-yGfp-ParBT1 |
| IVGB306 | PAO1 parST1-PA0290(327-R) tetO-PA0572(628-R) + pPSV35Ap-TetR-Cfp-yGfp-ParBT1 |
| IVGB307 | PAO1 parST1-PA2910(2,250-R) tetO-PA2523(2,672-R) + pPSV35Ap-TetR-Cfp-yGfp-ParBT1 |
| IVGB308 | PAO1 parST1-PA1643(2,538-L) tetO-PA0981(1,812-L) + pPSV35Ap-TetR-Cfp-yGfp-ParBT1 |
| **IVGB204** | **PAO1 PA1532(dnaX)-cfp parST1 PA2319(2,957-R) + pPSV35Ap-TetR-Cfp-yGfp-ParBT1** |
| **IVGB344** | PAO1 Δ*parA* parST1-PA2127(3,090-L) tetO-PA0069(82-R) + pPSV35Ap-TetR-Cfp-yGfp-ParBT1 |
| **IVGB359** | PAO1 Δ*parB* parST1-PA2127(3,090-L) tetO-PA0069(82-R) + pPSV35Ap-TetR-Cfp-yGfp-ParBT1 |
