# Supplementary material for: Chromosomal Organization and Segregation in Pseudomonas aeruginosa
Source: PLoS Genet. 2013 May 2;9(5):e1003492. doi: 10.1371/journal.pgen.1003492 (PMC3642087; doi:10.1371/journal.pgen.1003492)
Supplement: Table S3 — Percentage of one-focus cells for each chromosomal locus for each cell type in oriented cells (related to Figure 2). (DOCX) [file pgen.1003492.s009.docx]

**Table S3 :** Percentage of one-focus cells for each chromosomal locus for each cell type in oriented cells (related to Figure 2)

|  | **82-R** | **327-R** | **628-R** | **1,006-R** | **1,509-R** | **2,000-R** | **2,250-R** | **2,499-R** | **2,672-R** | **2,957-R** | **3,090-L** | **2,784-L** | **2,538-L** | **2,302-L** | **1,812-L** | **1,275-L** | **851-L** | **488-L** | **92-L** |
| --- | --- | --- | --- | --- | --- | --- | --- | --- | --- | --- | --- | --- | --- | --- | --- | --- | --- | --- | --- |
| **Proportion of 1-focus cells in small cells** | 21 | 38 | 61 | 84 | 87 | 100 | 100 | 100 | 100 | 100 | 100 | 100 | 100 | 100 | 96 | 84 | 67 | 37 | 28 |
| **Proportion of 1-focus cells in medium cells** | 0 | 0 | 4 | 23 | 30 | 86 | 90 | 97 | 100 | 100 | 100 | 100 | 97 | 92 | 54 | 23 | 6 | 0 | 0 |
| **Proportion of 1-focus cells in large cells** | 0 | 0 | 0 | 0 | 0 | 46 | 44 | 63 | 81 | 89 | 79 | 76 | 65 | 35 | 0 | 0 | 0 | 0 | 0 |
