# Supplementary material for: Chromosomal Organization and Segregation in Pseudomonas aeruginosa
Source: PLoS Genet. 2013 May 2;9(5):e1003492. doi: 10.1371/journal.pgen.1003492 (PMC3642087; doi:10.1371/journal.pgen.1003492)
Supplement: Text S1 — Supporting Materials and Methods. (DOCX) [file pgen.1003492.s010.docx]

**Text S1**

**Supporting Materials and Methods**

**Plasmids and Strains**

*Escherichia coli* DH5alpha (Invitrogen) was used as the recipient strain for all plasmid constructions, whereas *E. coli* strain β2163 {Demarre, 2005 #261} was used to mate plasmids into *P. aeruginosa*.

All the integration vectors carry the mobilization region from RP4, the ColE1 origin of replication, the *aacC1* gene (conferring resistance to gentamicin) and FRT sites for efficient Flp recombinase-mediated excision. They are derived from plasmid pP30D-FRT-YTAP {Vallet-Gely, 2005 #3}. pP30D-FRT-eGFP was constructed by replacing the DNA specifying the TAP-tag by DNA specifying the eGFP. Restriction sites (HindIII, PstI, KpnI and NotI) allow cloning of DNA fragments in frame with the eGFP. Similarly, pP30D-FRT-mcherry was constructed using DNA specifying mCherry from pROD17 (gift from C. Possoz), and pP30D-FRT-CFP was constructed using DNA specifying CFP. pP30D-FRT-parST1, which allows introduction of the *parS* sequence from plasmid pMT1 was obtained by cloning the EcoRI fragment containing the *parS* sequence from pGBKD3-parSpMT1 {Nielsen, 2006 #200} into the pP30D-FRT-mcherry plasmid, after digestion by MscI and XcmI, klenow treatment and religation. pP30D-FRT-tetO, allowing integration of tetO arrays, was constructed by replacing the EcoRI fragment from pP30D-FRT-parST1 by an EcoRI fragment from pFX240, carrying approximatively 140 *tetO* sequences and the *aacC1* gene {Lau, 2003 #202}.

Plasmid pPSV35Ap-TetR-Cfp-yGfp-ParBT1 is derived from pPSV35 {Rietsch, 2005 #4}. It carries the *bla* gene encoding the betalactamase, the PA origin of replication, *lacI^q^* and the isopropyl-D-thiogalactopyranoside (IPTG)-inducible lacUV5 promoter controlling the expression of genes encoding CFP tagged TetR and yGFP tagged ParB from the pMT1 plasmid. First, a XbaI/EcoRI fragment specifying the yGFP-ParBT1 from pFH 2923 {Nielsen, 2006 #200} was cloned into pPSV35. Then, a DNA fragment specifying TetR-CFP was amplified from pFX239 (gift from C. Possoz) using the PCR, and cloned using SacI/KpnI. Next, the *bla* gene was amplified using the PCR and cloned into the BglII site of the *aacC1* gene. Plasmid sequences are available on request.

In order to replace the chromosomal genes encoding HolB (PA2961), HolA(PA3898), HolC(PA3832) and DnaX (PA1532) by genes encoding eGFP tagged versions of these proteins, we engineered plasmids pP30D-FRT-2961-eGFP, pP30D-FRT-3989-eGFP and pP30D-FRT-1532-eGFP by cloning an approximately 600-bp fragment of DNA corresponding to a 3' portion of the *holB* gene and of the *holA* gene into HindIII-KpnI cut pP30D-FRT-eGFP, and an approximately 600-bp fragment of DNA corresponding to a 3' portion of the *dnaX* gene into HindIII-NotI cut pP30D-FRT-eGFP; the portion of both genes was cloned such that it was in-frame with the DNA specifying the eGFP protein. Strains PAO1 HolB-GFP, PAO1 HolA-GFP and PAO1 DnaX-GFP were constructed by mating PAO1 with β2163 containing pP30D-FRT-2961-eGFP, pP30D-FRT-3989-eGFP or pP30D-FRT-1532-eGFP and selected on *Pseudomonas* isolation agar (PIA) (Difco) containing gentamicin (60 μg/ml); because plasmids derived from pP30D-FRT-eGFP cannot replicate in *P. aeruginosa*, selection on PIA containing gentamicin results in isolation of those PAO1 cells in which the plasmid has integrated into the chromosome and, as a result, synthesize eGFP-tagged HolB, eGFP-tagged HolA, eGFP-tagged HolC or eGFP-tagged DnaX. We also cloned the 600-bp fragment of DNA corresponding to the 3' portion of the *dnaX* gene into HindIII-NotI cut pP30D-FRT-CFP, and used to it to construct the PAO1 DnaX-CFP strain, which synthesize CFP-tagged DnaX. We also replaced the NotI/BamHI fragment encoding the eGFP from plasmid pP30D-FRT-1532-eGFP by a NotI/BamHI fragment encoding the Dronpa protein to engineer the pP30D-FRT-1532-Dronpa plasmid. We then used this plasmid to construct the PAO1 DnaX-Dronpa strain, which synthesize Dronpa-tagged DnaX. Insertions were checked by the PCR. Growth rate was not significantly affected in these strains.

In order to integrate *parST1* sequences and *tetO* arrays at different positions of the *P. aeruginosa* genome, the same strategy was used: approximatively 500 bp of intergenic regions were cloned in pP30D-FRT-parST1 and pP30D-FRT-tetO, respectively. *P. aeruginosa* was mated with β2163 containing the resulting plasmids (Listed in Supplementary Table 1)*,* and transformants were selected on PIA supplemented with gentamicin. In order to introduce two chromosomal tags, the *parST1* sequence was first introduced at the desired location, and the plasmid backbone was excised through transient synthesis of FLP recombinase from plasmid pFLP2 (Hoang et al., 1998). Resulting strains were then mated with β2163 containing the pP30D-FRT-tetO derivatives and transformants were once again selected on PIA containing Gentamicin. In order to visualize the chromosomal tags, plasmid pPSV35Ap-TetR-Cfp-yGfp-ParBT1 was introduced by electroporation, and transformants selected on LB supplemented with Carbenicillin (300 μg/ml). Although strains with two chromosomal tags where sometimes slightly larger than wild type cells (by a maximum of 10%), their growth rate was not affected. Strains used in this study are listed in Supplementary Table 2.

The deletion constructs for the *parA* and the *parB* genes (PA5563 and PA5562 respectively) were generated by amplifying flanking regions by the PCR and then splicing the flanking regions together by overlap extension PCR; deletions were in-frame and contained the 6-bp linker sequence 5’-GAATTC-3’. The resulting PCR products were cloned on XbaI/HindIII fragments into plasmid pEXG2 (Rietsch et al., 2005), yielding plasmid pEXMparA and pEXMparB. These plasmids were then used to create strains PAO1 Δ*parA*, PAO1 Δ*parB*, PAO1 parST1-PA2127 Δ*parA* and PAO1 parST1-PA2127 Δ*parB* by allelic exchange. Deletions were confirmed by the PCR. The *tetO* arrays were subsequently inserted as described before.

**Supplemental references**

1. Demarre G*, et al.* (2005) A new family of mobilizable suicide plasmids based on broad host range R388 plasmid (IncW) and RP4 plasmid (IncPalpha) conjugative machineries and their cognate Escherichia coli host strains. *Research in microbiology* 156(2):245-255.

2. Vallet-Gely I, Donovan KE, Fang R, Joung JK, & Dove SL (2005) Repression of phase-variable cup gene expression by H-NS-like proteins in Pseudomonas aeruginosa. *Proceedings of the National Academy of Sciences of the United States of America* 102(31):11082-11087.

3. Nielsen HJ, Ottesen JR, Youngren B, Austin SJ, & Hansen FG (2006) The Escherichia coli chromosome is organized with the left and right chromosome arms in separate cell halves. *Molecular microbiology* 62(2):331-338.

4. Lau IF*, et al.* (2003) Spatial and temporal organization of replicating Escherichia coli chromosomes. *Molecular microbiology* 49(3):731-743.

5. Rietsch A, Vallet-Gely I, Dove SL, & Mekalanos JJ (2005) ExsE, a secreted regulator of type III secretion genes in Pseudomonas aeruginosa. *Proceedings of the National Academy of Sciences of the United States of America* 102(22):8006-8011.
